# Designing an AI-Responsive Early Childhood Curriculum: A Conceptual Framework for Supporting Developmental Learning Trajectories in Preschool Education

**DOI:** 10.12688/f1000research.185645.1

**Published:** 2026-07-16

**Authors:** Herlina Herlina, Chandra Apriyansyah, Rusmayadi Rusmayadi, Herman Herman

**Affiliations:** 1 Early Childhood Education Program, Universitas Negeri Makassar, Makassar, South Sulawesi, 90222, Indonesia; 2 Doctoral Program in Early Childhood Education, Universitas Negeri Jakarta, East Jakarta, DKI Jakarta, 13320, Indonesia

**Keywords:** Artificial Intelligence; Early Childhood Education; Curriculum Design; Deep Learning; Teacher Agency; AI-Responsive Curriculum Design (ARCD)

## Abstract

**Background:**

The rapid integration of Artificial Intelligence (AI) and deep learning technologies into educational systems has created new opportunities for personalized learning in early childhood education (ECE). Despite increasing interest in AI-enhanced pedagogy, limited research has examined how educators conceptualize and implement AI-responsive curricula, and no contextually grounded framework currently explains this process in preschool settings.

**Purpose:**

This study aims to explore educators’ experiences in designing and implementing AI-responsive curricula and to develop a conceptual framework that explains the integration of AI technologies into curriculum decision-making for young children.

**Method:**

An interpretive qualitative approach was employed at TK Harapan Bangsa, Makassar, Indonesia. Data were collected through phenomenological interviews with six preschool teachers and two curriculum specialists, classroom observations, and document analysis of AI-supported lesson-planning systems. Data were analyzed using thematic analysis guided by Vygotsky’s Zone of Proximal Development, Bronfenbrenner’s Ecological Systems Theory, and the Universal Design for Learning (UDL) framework.

**Findings:**

Three interconnected constructs emerged from the analysis. First, Predictive Developmental Tracking describes the use of AI to identify and anticipate children’s developmental readiness across learning domains. Second, AI-Responsive Curriculum Adaptation reflects a dynamic process in which teachers evaluate algorithmic recommendations and align them with contextual classroom needs. Third, Deep Learning as Pedagogical Scaffold positions AI as a supportive tool that strengthens, rather than replaces, professional teacher judgment. These constructs informed the development of the AI-Responsive Curriculum Design (ARCD) Framework, which illustrates how predictive analytics, curriculum adaptation, and teacher expertise interact to support individualized developmental learning trajectories.

**Conclusion:**

The study contributes a novel ARCD Framework that explains AI-responsive curriculum implementation in ECE. The findings highlight the importance of balancing technological intelligence with pedagogical expertise and provide implications for curriculum policy, teacher professional development, and ethical AI integration in early childhood education.

## Introduction

The twenty-first century has ushered in an unprecedented convergence of educational science and artificial intelligence (AI), fundamentally redefining what is possible within early childhood education (ECE) settings. Global educational systems increasingly recognize that the earliest years of learning spanning birth to six years represent the most neuroplastic and formative period of human development ( Shonkoff & Phillips, 2020). Concurrently, the proliferation of deep learning technologies, machine learning algorithms, and adaptive educational systems has ignited scholarly and practitioner interest in whether these tools can meaningfully support individualized developmental pathways for young learners ( Holmes et al., 2022; Luckin et al., 2023).

Within this dynamic landscape, two distinct scholarly trajectories have developed in relative isolation. The first concerns AI and machine learning applications in formal schooling primarily at the secondary and tertiary levels focusing on performance prediction, learning analytics, and adaptive content delivery ( Zawacki-Richter et al., 2019). The second pertains to developmentally appropriate practice in ECE, which emphasizes play-based learning, holistic development, and the central role of educator judgment and relational pedagogy ( NAEYC, 2020; Fleer, 2021). The intersection of these two domains specifically, the design of AI systems that are genuinely responsive to the fluid, multimodal, and emergent nature of early childhood development remains theoretically underdeveloped and empirically underexplored ( Véliz et al., 2024).

This gap is both consequential and urgent. As governments and education ministries globally invest in AI-integrated learning platforms for preschool children, the risk of deploying technologically sophisticated but developmentally inappropriate tools becomes acute ( UNESCO, 2023). Current AI educational tools, predominantly designed using large-scale quantitative datasets from older learners, frequently fail to account for the qualitative complexity of early childhood developmental trajectories the non-linearity, the domain interdependencies, and the profound influence of socio-cultural context on learning readiness ( Pea, 2022; Rogoff, 2023). Without a robust theoretical and empirical framework to guide their design, these tools risk replicating existing educational inequities at scale.

The Indonesian preschool context provides a particularly instructive case study for examining this intersection. Indonesia’s national preschool curriculum (Kurikulum PAUD) emphasizes holistic child development across cognitive, language, social-emotional, physical-motor, and value-moral domains ( Kemendikbud, 2022). However, the rapid governmental push toward digitalization in education most prominently through the Merdeka Belajar (Liberated Learning) initiative has created an implementation tension: educators are increasingly expected to leverage digital and AI-based tools while navigating the profound developmental heterogeneity of their preschool classrooms ( Halim & Wahyuni, 2024).

Against this backdrop, this study investigates how AI-responsive curricula can be conceptualized and implemented to support individualized developmental learning trajectories in preschool education. The study is grounded in a qualitative, phenomenological inquiry, prioritizing the lived experiences, professional judgments, and interpretive meaning-making of ECE educators and curriculum specialists as the primary epistemic lens through which AI integration is understood. Rather than evaluating algorithmic performance metrics, this research asks how educators negotiate the introduction of AI tools within the relational and developmental complexities of early childhood classrooms at a single pilot school in Indonesia, with findings intended to inform broader contextually-sensitive implementations.

Theoretically, the study is anchored in three complementary frameworks. First, Vygotsky’s Zone of Proximal Development (ZPD) ( Vygotsky, 1978) provides a conceptual foundation for understanding AI as a potential scaffold for developmental readiness a “more knowledgeable other” operating in the digital domain. Second, Bronfenbrenner’s Ecological Systems Theory (1979) situates the child’s developmental trajectory within layered socio-ecological contexts, reminding us that any AI-responsive curriculum must be sensitive to family, community, and cultural dimensions of learning. Third, the Universal Design for Learning (UDL) framework ( CAST, 2018) offers a commitment to equitable, flexible curriculum design that accommodates the full spectrum of early childhood developmental variability.

This research addresses two primary questions: (1) How do ECE educators conceptualize and negotiate the integration of AI-responsive systems into preschool curriculum design? and (2) What emergent principles characterize an effective AI-Responsive Curriculum Design (ARCD) framework for supporting developmental learning trajectories in early childhood settings?

The following sections present a comprehensive literature review situating AI in ECE, a rigorous qualitative methodology, rich phenomenological findings, and a discussion that synthesizes these insights into a theoretically grounded and practically transferable ARCD framework.

## Literature review

### Artificial intelligence and deep learning in educational contexts

The application of artificial intelligence in formal education has accelerated dramatically over the past decade, driven by advancements in machine learning, natural language processing, and neural network architectures. Holmes et al. (2022) provide a comprehensive taxonomy of AI in education (AIED), categorizing tools into three functional types: *predictive systems* (which forecast learner performance), *adaptive systems* (which adjust content delivery in real time), and *generative systems* (which produce novel learning materials). Among these, deep learning architectures have demonstrated promise for processing complex, sequential, and multimodal data generated by learners during structured activities ( LeCun et al., 2015; Goodfellow et al., 2016). More recently, the emergence of generative AI, large language models (LLMs), and foundation models has expanded these possibilities further, enabling more flexible and context-sensitive educational applications ( Brown et al., 2020; Bommasani et al., 2021; Kasneci et al., 2023). Explainable AI (XAI) frameworks have also emerged as a critical development, enabling educators to interrogate algorithmic decision-making processes rather than accepting opaque outputs ( Arrieta et al., 2020; Khosravi et al., 2022).

In the context of learning trajectory prediction, deep learning models have been applied to identify patterns in learner interaction data that are imperceptible to human observers ( Pérez-Álvarez et al., 2022). Luckin et al. (2023) argue that such systems, when ethically designed and educator-informed, hold the potential to democratize personalized learning by providing teachers with actionable intelligence about individual developmental needs. However, these scholars also caution that the deployment of AIED tools without robust pedagogical frameworks risks reducing complex developmental phenomena to algorithmic proxies, particularly in early childhood contexts where developmental trajectories are highly individualized and culturally situated.

### Early childhood development and the limitations of current AI tools

Early childhood development is characterized by domain interdependence, non-linearity, and profound sensitivity to environmental and relational contexts ( Shonkoff & Phillips, 2020). The five developmental domains central to Indonesian ECE cognitive, language-literacy, social-emotional, physical-motor, and value-moral do not develop in isolation; progress in one domain frequently enables or constrains growth in another ( Kemendikbud, 2022). This systemic, emergent quality of early childhood development poses a fundamental challenge for AI tools designed using reductive performance metrics.

Véliz et al. (2024) conducted a systematic review of AI applications in ECE across eighteen countries, finding that 73% of existing AI tools were designed primarily to measure cognitive or literacy outcomes, with minimal attention to social-emotional or value-moral development. Fleer (2021) argues that this cognitive reductionism reflects a broader epistemological bias in AIED research toward measurable, linear learning outcomes that align more readily with algorithmic modeling but poorly represent the holistic developmental reality of young children. Similarly, Rogoff (2023) emphasizes that developmental trajectories are culturally participatory processes, meaning that any AI system aspiring to predict them must incorporate cultural context variables—a design requirement largely absent from current commercial ECE platforms.

### Vygotsky’s zone of proximal development and AI scaffolding

Vygotsky’s (1978) concept of the Zone of Proximal Development (ZPD) offers a powerful theoretical lens for conceptualizing the role of AI in early childhood learning. The ZPD describes the developmental space between what a child can accomplish independently and what becomes possible with knowledgeable support. Traditionally, this support has been provided by a teacher, parent, or more capable peer; however, emerging scholarship suggests that well-designed AI systems can function as digital scaffolds within this zone, providing responsive, individualized support calibrated to real-time developmental assessments ( Pea, 2022).

Palincsar and Brown’s (1984) foundational work on scaffolded instruction, extended by Wood et al. (1976), identifies key scaffold characteristics contingency, fading, and intentionality that are directly applicable to the design of AI-responsive learning systems. A deep learning framework capable of predicting a child’s developmental trajectory must not only identify current competency levels but also anticipate the proximal zone of achievable growth, thereby informing curriculum adaptations that are neither too simple (producing boredom) nor too complex (producing frustration) ( Luckin et al., 2023).

### Bronfenbrenner’s ecological systems theory and culturally situated AI

Bronfenbrenner’s (1979) Ecological Systems Theory situates child development within concentric, interacting environmental systems the microsystem (immediate family and classroom), mesosystem (school-family interface), exosystem (community and institutional policies), and macrosystem (cultural values and societal structures). Applied to AI-responsive curriculum design, this framework insists that developmental learning trajectory predictions cannot be meaningful without incorporating data from multiple ecological layers. A child’s trajectory in language literacy, for example, cannot be accurately predicted by classroom interaction data alone; family language practices, community linguistic norms, and the socioeconomic resources of the home environment are equally determinative ( Rogoff, 2023).

Recent scholarship on culturally responsive AI in education ( Gay, 2018; Paris, 2012; Gonzalez et al., 2023) reinforces this ecological imperative by demonstrating that AI systems trained on culturally hegemonic datasets systematically underpredict the developmental potential of children from marginalized or indigenous communities. In the Indonesian context, this concern is particularly salient given the country’s extraordinary linguistic and cultural diversity across more than 700 ethnic groups ( Sakhiyya et al., 2025).

### Universal design for learning and equitable AI curriculum design

The Universal Design for Learning (UDL) framework ( CAST, 2018; Rose & Meyer, 2022) provides a third theoretical pillar for this study. UDL posits that effective curriculum design must offer multiple means of representation, action and expression, and engagement—proactively eliminating barriers to learning rather than retrofitting accommodations for learners who do not fit dominant templates. Applied to AI-responsive ECE curricula, UDL principles demand that deep learning systems be designed to surface and respond to developmental variability as a feature rather than a deficiency, providing adaptive pathways that honor the full spectrum of preschool developmental trajectories.

Critically, Tomlinson and Imbeau (2023) and Waitoller and King Thorius (2022) both caution that without intentional equity centering in design, AI-responsive tools risk codifying existing developmental biases systematically identifying children from dominant cultural and socioeconomic groups as “on-trajectory” while flagging children from marginalized communities as “at-risk,” thereby deepening structural inequities under the guise of objectivity.

### Gaps in the literature and research rationale

Despite the growing body of literature at the intersection of AI and ECE, three critical gaps remain unaddressed. First, there is a near-total absence of qualitative inquiry into educator experiences of AI-responsive curriculum integration in preschool settings; the dominant methodological paradigm remains quantitative performance measurement ( Zawacki-Richter et al., 2019). Second, existing frameworks for AI in education rarely address the developmental, ecological, and cultural complexity specific to early childhood, instead adapting frameworks from K–12 or higher education contexts ( Véliz et al., 2024). Third, and most critically, no validated framework exists for designing AI-responsive curricula that specifically target the prediction and support of developmental learning trajectories across all five early childhood developmental domains in culturally diverse preschool contexts. This study directly addresses these gaps.

## Methodology

### Research design and paradigm

This study adopted a qualitative, phenomenological research design grounded in the interpretivist paradigm. Phenomenology particularly the hermeneutic tradition associated with Heidegger (1962) and Gadamer (1975) and its educational applications as articulated by van Manen (2016) was selected as the most appropriate design because the central research questions concern the *lived experience* of educators navigating AI integration in early childhood settings. Phenomenology privileges the subjective, meaning-laden dimensions of human experience, making it ideally suited to capture the nuanced professional interpretations and pedagogical negotiations that constitute curriculum design in practice ( Creswell & Poth, 2018).

This study explicitly does not employ experimental or quasi-experimental methods, as its aim is not to measure algorithmic performance metrics or compare AI-assisted and non-AI-assisted learning outcomes. Rather, the goal is to construct a theoretically grounded and empirically rich understanding of how AI-responsive curricula can be conceptualized and designed in a manner that is genuinely responsive to early childhood developmental complexity.

### Research setting

The research was conducted at TK Harapan Bangsa, a public preschool located in the Tamalanrea district of Makassar, South Sulawesi, Indonesia. This setting was selected for three reasons: (1) it serves a socioeconomically diverse student population representative of the broader urban ECE landscape in Eastern Indonesia; (2) the school has been a pilot site for the Merdeka Belajar digital integration initiative since 2023, providing an active context of AI tool engagement; and (3) the school’s leadership demonstrated a strong commitment to reflective practice and teacher professional development, ensuring rich qualitative data generation.

### Participants

Participants were selected through purposive sampling ( Patton, 2015) to ensure maximum variation in professional experience, developmental domain specialization, and degree of prior AI engagement. The final participant group consisted of:

1. **ECE Classroom Educators (n = 6):** Teachers with between two and fifteen years of experience in preschool settings, representing the full range of developmental domain expertise. All held at minimum a Diploma IV or Bachelor’s degree in Early Childhood Education.
2. **Curriculum Specialists (n = 2):** Senior ECE curriculum developers affiliated with the Dinas Pendidikan Kota Makassar with expertise in integrating national curriculum standards with technology-enhanced learning environments.
3. **AI Tool Developer-Collaborator (n = 1):** An educational technology specialist involved in the design of the AI platform piloted at the school, who participated in a single extended expert interview to provide technical grounding for the educator accounts.

Prior to data collection, both written and verbal informed consent were obtained from all participants. Verbal consent was employed as an additional measure to ensure that participants fully understood the study’s objectives, procedures, potential benefits and risks, and their rights as research participants. This approach was adopted to enhance participant comfort and to facilitate direct clarification of any questions before the interviews commenced. Ethical clearance was granted by the Research Ethics Committee of Universitas Negeri Makassar (Protocol No. 2024-ECE-AI-07). Participation was entirely voluntary, and participants were informed of their right to decline answering any question or to withdraw from the study at any stage without any consequences. All data were anonymized using coded identifiers to protect participant confidentiality and privacy and were securely stored in accordance with established research ethics standards.

Table 1 presents the demographic characteristics of the nine study participants involved in the development of the AI-Responsive Curriculum Design (ARCD) framework. The participant group was predominantly male (67%, *n* = 6), while female participants accounted for 33% ( *n* = 3). In terms of professional roles, most participants were early childhood education (ECE) classroom educators (67%, *n* = 6), complemented by curriculum specialists (22%, *n* = 2) and one AI tool developer-collaborator (11%, *n* = 1), ensuring a diverse range of pedagogical and technological perspectives. Professional experience was evenly distributed, with one-third of participants reporting 1–5 years, 6–10 years, and more than 10 years of experience, respectively. Regarding AI integration experience, the majority were novice users with less than one year of experience (44%, *n* = 4), followed by intermediate users with one to two years of experience (33%, *n* = 3) and experienced users with more than two years of AI integration experience (22%, *n* = 2). This participant composition provided a balanced representation of educational expertise, curriculum leadership, and technological knowledge, enabling a comprehensive exploration of how AI-responsive curricula are conceptualized and implemented within early childhood education settings.

**Table 1.** Demographic profile of study participants.

Characteristic	Category	Frequency (n)	Percentage (%)
Gender	Male	6	67%
	Female	3	33%
Participant Role	ECE Classroom Educator	6	67%
	Curriculum Specialist	2	22%
	AI Tool Developer-Collaborator	1	11%
Professional Experience	1–5 Years	3	33%
	6–10 Years	3	33%
	> 10 Years	3	33%
AI Integration Experience	Novice (< 1 Year)	4	44%
	Intermediate (1–2 Years)	3	33%
	Experienced (> 2 Years)	2	22%

### Data Collection Procedures

Data were collected through three complementary instruments, ensuring methodological triangulation ( Denzin, 2012) across multiple sources of evidence:

1. **Semi-Structured Phenomenological Interviews.** Each educator participant engaged in two in-depth interviews conducted over the twelve-week fieldwork period. Interviews averaged 65 minutes in duration and were audio-recorded with participant consent. Interview questions were designed to elicit rich experiential accounts across three thematic domains: (a) educators’ initial encounters with and conceptualizations of AI-responsive tools; (b) their moment-to-moment negotiation of algorithmic suggestions during curriculum planning; and (c) their interpretations of how AI-generated developmental trajectory data influenced their relational and pedagogical decision-making with individual children. All interviews were conducted in Bahasa Indonesia and subsequently transcribed verbatim.
2. **Non-Participant Classroom Observations.** Eighteen classroom observation sessions were conducted across the six educator participants, each lasting 45 minutes, over the twelve-week period. Observations focused on educator-AI-child interaction sequences: specifically, instances where educators consulted AI-generated trajectory data during lesson transitions, used algorithmic suggestions to differentiate activities, or made explicit professional judgments that departed from or refined AI recommendations. A structured observation protocol was employed, supplemented by detailed field notes capturing the relational and affective dimensions of classroom dynamics.
3. **Document Analysis.** Relevant curricular and planning documents were collected and analyzed, including AI-generated weekly learning trajectory reports, educator reflective journals, lesson plan adaptations, and parent communication records. Document analysis followed Bowen’s (2009) framework for systematic documentary evidence, attending to the purposes, audiences, and institutional contexts of each document type.
  All data collection procedures were conducted in accordance with the ethical principles outlined in the Declaration of Helsinki and the American Educational Research Association’s (AERA) Code of Ethics (2011). Children present in the observed classrooms were not research subjects; they were present as part of the natural classroom environment, and no identifying information about individual children was collected.

### Data analysis

Data analysis followed Giorgi’s (2009) systematic phenomenological method, adapted for educational research contexts by Creswell and Poth (2018). The analytical process proceeded through four sequential and iterative stages:

**Stage 1 Horizonalization:** All interview transcripts, observation notes, and documentary excerpts were read repeatedly in their entirety to achieve a global sense of the data. Relevant statements pertaining to educators’ experiences of AI integration were identified and listed as horizons of equal significance, without preliminary hierarchical ordering.

**Stage 2 Reduction and Clustering:** Horizons were grouped into meaning units based on thematic overlap and conceptual proximity. Redundant and overlapping statements were eliminated, and each meaning unit was transformed into a phenomenological expression a statement that captures the essence of the experience without imposing interpretive distortion.

**Stage 3 Imaginative Variation:** The structural dimensions of each meaning unit were explored by systematically varying the conditions under which the experience might occur, enabling the identification of invariant structural features of the phenomenon. This stage involved extensive reflexive memo-writing to distinguish between features of the experience that were contextually contingent and those that appeared essential to the phenomenon itself.

**Stage 4 Synthesis of Textural and Structural Descriptions:** The final synthesis integrated the textural description (what educators experienced) with the structural description (how they experienced it), producing a composite phenomenological description of the experience of designing and implementing AI-responsive curricula in early childhood settings.

The qualitative data management and analysis were supported by MAXQDA 2024 software. To ensure analytical rigor, a full audit trail of reflexive memos, analytical decisions, and emergent code frameworks was maintained throughout the study. Member checking was conducted at the conclusion of the analysis phase: a summary of emergent constructs and their supporting evidence was shared with four of the six educator participants, all of whom confirmed the resonance and accuracy of the interpretations. Peer debriefing was conducted with two senior colleagues external to the study to challenge analytical assumptions and reduce interpretive bias.

Figure 1 illustrates the conceptual architecture of the **AI-Responsive Curriculum Design (ARCD) Framework**, which operates through three interconnected layers: input, processing, and output. The first layer focuses on **child developmental data collection**, incorporating multiple sources of evidence such as observational notes, portfolios, assessment records, and play-based developmental indicators. These data are subsequently analyzed within the second layer through **deep learning algorithms and AI-responsive processing**, including pattern recognition, temporal analysis, and multi-domain data integration, enabling the identification of developmental trends and individualized learning needs. The third layer translates these analytical insights into practical educational outcomes, including **personalized learning trajectories**, adaptive curriculum recommendations, teacher feedback, and parent progress reports. This framework conceptualizes AI not as a replacement for educators but as a decision-support system that transforms diverse developmental data into actionable pedagogical insights, thereby facilitating more responsive, individualized, and evidence-based curriculum implementation in early childhood education settings.

**Figure 1.**
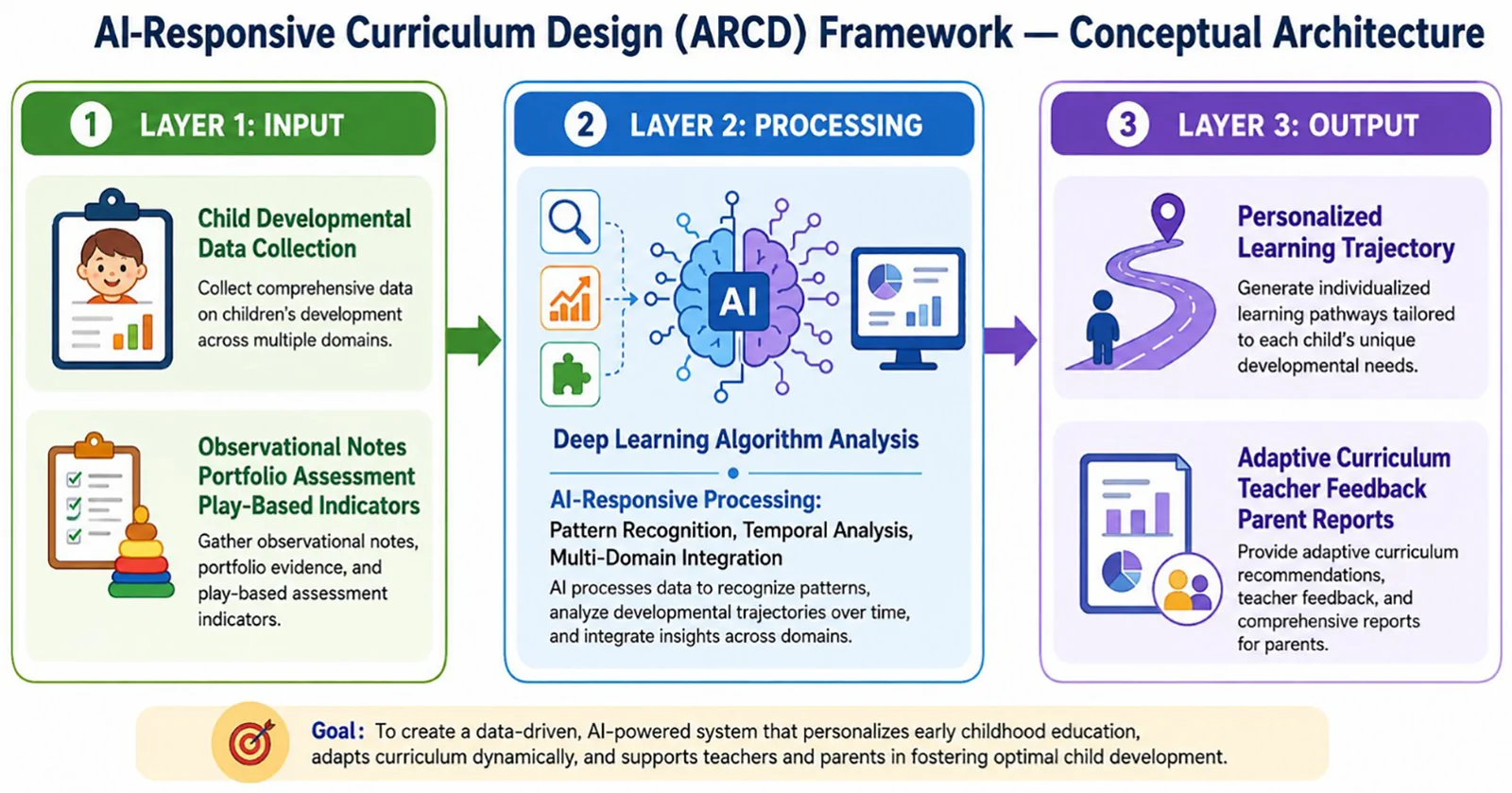
AI-Responsive Curriculum design (ARCD) framework — Conceptual architecture.

## Findings and discussion

Data analysis generated three primary emergent constructs, each representing a distinct dimension of educators’ phenomenological experience of designing and implementing AI-responsive curricula in their preschool classrooms. These constructs are presented below with rich evidential grounding from the phenomenological data, followed by an integrated discussion linking each to the theoretical frameworks anchoring this study.

### Construct 1: Predictive Developmental Tracking: AI as Early Warning System

The first and most consistently articulated emergent construct across all educator participants concerned the phenomenon of *Predictive Developmental Tracking* the experience of AI-generated trajectory data surfacing developmental concerns, patterns, or readiness indicators that had not yet become perceptible to the educator through conventional observational means. Educators described a distinctive phenomenological quality to this experience: a sense of cognitive dissonance followed by professional recalibration, wherein algorithmic insights temporarily destabilized their existing developmental assumptions about individual children, ultimately enriching their pedagogical responsiveness.

One educator with eleven years of classroom experience described this experience in the following terms:

*“When the AI flagged that Rafi was showing micro-patterns of reduced engagement in spatial reasoning tasks specifically a 23% decline in interaction duration across three consecutive sessions I initially dismissed it. I thought, ‘I know this child; he’s fine.’ But when I went back and reviewed my own observation notes from those same sessions, I could see in retrospect what the algorithm had detected in real time. That moment changed how I relate to the data. It’s not replacing my observation it’s sharpening it.”* (Educator P1, Interview 2, Week 9).

This account illustrates the central structural feature of Predictive Developmental Tracking: the AI system’s capacity to detect developmental signals across temporal sequences of behavioral data that exceed the perceptual bandwidth of individual educators managing full classrooms of 15 to 20 children. Across all participant accounts, this capacity was described not as a source of anxiety or professional threat, but as a form of *amplified perception* a phenomenological extension of the educator’s observational capacity into temporal and multimodal data dimensions that are ordinarily invisible to classroom practice.

Theoretically, this construct resonates with and extends Vygotsky’s (1978) concept of the ZPD in a novel direction. Just as ZPD describes the developmental space made accessible through knowledgeable scaffolding for the child, these data suggest the existence of a parallel *Educator ZPD* a professional developmental space that AI systems can expand by providing teachers with access to developmental intelligence that lies beyond their unaided perceptual reach. This interpretation extends Pea’s (2022) theorization of distributed cognition in educational technology, suggesting that AI tools function most effectively when they are conceptualized as cognitive prosthetics that enhance professional agency rather than systems that replace professional judgment.

The curriculum specialist participants provided a complementary institutional perspective on Predictive Developmental Tracking. Both specialists described experiences in which AI-generated trajectory data had identified systematic developmental disparities across the school’s preschool population that had not been captured by existing assessment instruments:

*“The system generated a report showing that children from bilingual Bugis-Indonesian households were systematically showing delayed trajectory predictions in early literacy not because they were cognitively behind, but because the AI’s literacy indicators were calibrated exclusively to Bahasa Indonesia benchmarks. We used that finding to redesign the tracking variables. The AI became a mirror that showed us our own curriculum’s cultural assumptions.”* (Curriculum Specialist CS1, Interview 1, Week 4).

This insight demonstrates the potential of Predictive Developmental Tracking to function as an equity-revealing mechanism surfacing systemic biases in developmental benchmarking that might otherwise remain institutionally invisible. This finding resonates with Waitoller and King Thorius’s (2022) critique of deficit-oriented developmental assessment and supports Paris’s (2012) call for culturally sustaining practices that honor linguistic and cultural diversity as developmental assets rather than deficits.

As presented in Table 2, the qualitative analysis process transformed raw observational and interview data into higher-order conceptual constructs through open, axial, and selective coding procedures. The findings reveal four interconnected emergent concepts that characterize AI-responsive curriculum implementation in early childhood education. First, Predictive Developmental Tracking emerged from observations showing that AI systems could identify potential developmental changes before they became visible to teachers, functioning as an early-warning mechanism for developmental monitoring. Second, AI-Responsive Curriculum Adaptation reflected the capacity of AI-supported systems to dynamically adjust learning content and instructional sequences based on real-time developmental data and learning progress. Third, Deep Learning as Pedagogical Scaffold highlighted the role of AI in strengthening teacher practices by facilitating evidence-based communication with parents and supporting pedagogical decision-making rather than replacing teacher expertise. Finally, Equity-Centered Developmental Prediction demonstrated how algorithmic insights could uncover previously overlooked developmental potential, enabling more inclusive and individualized learning opportunities for children who might otherwise be underestimated. Collectively, these findings indicate that AI functions not merely as a technological tool but as an adaptive pedagogical partner that enhances developmental assessment, curriculum responsiveness, teacher agency, and educational equity within early childhood learning environments.

**Table 2.** Data abstraction matrix: From raw observation to emergent construct.

Open coding (Raw Observation)	Axial coding (Sub-Theme)	Selective coding (Emergent Concept)
“Teacher observes that the AI system flagged a child’s reduced fine motor engagement before the teacher noticed any change.” (Field Note, Week 3)	AI as early-warning system for developmental regression	Predictive Developmental Tracking
“The curriculum module automatically shifted from visual-spatial activities to language-rich tasks after detecting a plateau in the child’s pattern recognition scores.” (Observation Log, Week 5)	Adaptive content sequencing based on real-time learning data	AI-Responsive Curriculum Adaptation
“Educator noted that the AI-generated trajectory report helped her discuss each child’s development in parent conferences with more specific evidence.” (Interview, Week 8)	AI as mediator of teacher–parent communication	Deep Learning as Pedagogical Scaffold
“A child who was previously labeled as ‘slow’ showed advanced symbolic reasoning once the AI recommended play-based mathematical provocations.” (Teacher Reflective Journal, Week 10)	Reclassification of developmental potential through algorithmic insight	Equity-Centered Developmental Prediction

### Construct 2: AI-Responsive curriculum adaptation - Negotiating algorithm and pedagogy

The second emergent construct, *AI-Responsive Curriculum Adaptation*, describes the complex, iterative process through which educators negotiated between AI-generated curricular suggestions and their contextual professional knowledge of individual children, classroom dynamics, and family circumstances. Far from a passive acceptance of algorithmic recommendations, this process was characterized by active, reflective dialogue between the educator and the system—a dynamic that all participants described as requiring a new form of pedagogical literacy that had not been part of their pre-service or in-service professional training.

Educators consistently described a tripartite cognitive process in their curriculum adaptation negotiations: first, *algorithmic reception* (receiving and initially interpreting the AI-generated trajectory suggestion); second, *contextual interrogation* (actively questioning the suggestion in light of their relational and situational knowledge of the child’s family background, emotional state, and recent classroom experiences); and third, *professional synthesis* (arriving at a curriculum adaptation that integrated algorithmic intelligence with contextual pedagogical judgment). This tripartite process was described with particular clarity by one educator:

*“The AI told me Sari needed more fine motor challenges—cutting, threading, building. But I knew that last week her parents had separated, and she was coming to school visibly distressed. The algorithm doesn’t know that. So I took the AI’s developmental logic and applied it through an emotional lens—I introduced sensory play activities that built fine motor capacity while also providing calming, self-regulatory experiences. That’s not the algorithm; that’s me using the algorithm.”* (Educator P4, Interview 2, Week 7).

This account captures what became the defining phenomenological quality of AI-Responsive Curriculum Adaptation across all participants: the insistence that effective AI-responsive curriculum design is not a *substitution* of human professional judgment by algorithmic calculation, but rather a *dialogic synthesis* in which the educator retains ultimate interpretive authority while gaining expanded developmental intelligence through the AI system. This finding resonates with and extends Gay’s (2018) framework of Culturally Responsive Teaching: just as culturally responsive pedagogy requires teachers to actively integrate students’ cultural realities into learning design, AI-responsive curriculum adaptation requires teachers to actively integrate the child’s relational, emotional, and ecological realities into algorithmic developmental recommendations.

The AI Tool Developer-Collaborator provided an important technical dimension to this construct, describing how the AI system’s design philosophy was intended to support rather than determine educator decision-making:

*“We deliberately designed the system to present trajectory predictions as probability ranges rather than definitive assessments precisely because we knew that the contextual variables educators carry in their heads are irreducibly important. A 73% probability that a child will benefit from increased spatial reasoning challenges should be read as an invitation to investigate, not an instruction to execute.”* (AI Developer D1, Expert Interview, Week 6).

Theoretically, the construct of AI-Responsive Curriculum Adaptation provides robust empirical support for and extension of the UDL framework ( CAST, 2018). The UDL principle of *multiple means of action and expression* here finds a novel instantiation: the AI-responsive curriculum itself becomes a multiply-expressive system, offering educators probabilistic developmental intelligence that they can enact through a diverse repertoire of pedagogical approaches calibrated to each child’s ecological and relational context. The construct also advances Tomlinson and Imbeau’s (2023) theory of differentiated instruction by demonstrating that AI trajectory data can serve as a dynamic differentiation scaffold expanding the evidence base from which teachers make curricular adaptation decisions without collapsing the irreducibly interpretive nature of that decision-making process.

### Construct 3: Deep learning as pedagogical scaffold — AI Amplifying teacher expertise

The third and most theoretically generative emergent construct, *Deep Learning as Pedagogical Scaffold*, represents the overarching experiential meaning that educators attributed to their sustained engagement with the AI-responsive curriculum system over the twelve-week period. In contrast to initial apprehensions about AI as a potential threat to professional autonomy apprehensions that were expressed by four of the six educator participants during the first interview session—all participants described a progressive phenomenological reorientation toward the system as a form of professional amplification: a tool that enhanced rather than diminished their capacity for relational, responsive, and developmentally appropriate pedagogy.

This reorientation was described with particular depth by the most experienced educator participant (15 years of classroom practice), who initially expressed the strongest reservations about AI integration:

*“In the beginning, I thought, ‘This machine is going to tell me how to teach.’ But over the weeks, I began to see it differently. The AI doesn’t know my children it knows patterns in their data. I know my children. What happens when you put those two kinds of knowing together is something more powerful than either one alone. It’s like having a very data-literate teaching assistant who never forgets anything and who reminds you to look at things you’ve unconsciously stopped noticing.”* (Educator P2, Interview 2, Week 11).

This account crystallizes the central phenomenological insight of this construct: effective AI integration in early childhood curriculum design involves a relational epistemology a form of professional knowledge that is constituted through the productive dialogue between *algorithmic knowing* (pattern recognition across large developmental datasets) and *relational knowing* (the contextual, empathic, and culturally sensitive professional intelligence of the experienced educator). Neither form of knowing is sufficient alone; it is their synthesis that generates the enhanced pedagogical capacity that participants consistently described as the most significant outcome of their AI engagement.

This construct provides a significant empirical extension to Wood et al.’s (1976) theory of scaffolded instruction. In the original formulation, scaffolding describes the support provided by a more knowledgeable human partner that enables a learner to accomplish tasks beyond their independent capability. The data from this study suggest that deep learning frameworks can function as scaffolding not only for children’s learning by predicting and supporting developmental readiness but for *educators’ professional practice*: by expanding the developmental intelligence available to teachers, AI systems can scaffold educators toward higher orders of pedagogical responsiveness than they could achieve through unaided professional knowledge alone.

Critically, all participants emphasized that this scaffold function was conditional: it depended on AI systems being designed with transparency (providing educators with intelligible explanations of algorithmic reasoning), humility (presenting predictions as probabilistic rather than definitive), and equity-sensitivity (actively accounting for cultural and linguistic variables in developmental trajectory modeling). When these conditions were absent as described by two participants who had prior experience with less sophisticated commercial AI platforms the scaffolding function collapsed, and the AI became an authoritarian instrument that suppressed rather than amplified professional judgment.

As shown in Table 3, the proposed AI-responsive curriculum framework maps multiple developmental domains to specific AI-generated indicators, corresponding data sources, and projected developmental trajectories. The cognitive domain is assessed through pattern recognition speed derived from digital game interaction logs, producing indicators of problem-solving readiness. In the language and literacy domain, vocabulary production rates extracted through AI-based audio transcription systems are used to predict children’s early literacy trajectories. Social-emotional development is monitored through peer interaction frequency obtained from video observation coding, generating measures of self-regulation maturity. Physical-motor development is represented by fine and gross motor precision captured through wearable sensor data, which informs individualized motor development pathways. Meanwhile, creative expression is assessed through divergent play episodes documented in portfolio analyses, enabling projections of children’s creative capacities. Collectively, these findings demonstrate how AI systems can integrate multimodal developmental data to generate individualized learning trajectories across cognitive, linguistic, social-emotional, motor, and creative domains, thereby supporting more responsive, evidence-based curriculum planning in early childhood education settings.

**Table 3.** Developmental domain mapping: AI indicator variables and trajectory outputs.

Developmental domain	AI indicator variable	Data source	Trajectory output
Cognitive	Pattern recognition speed	Digital game interaction logs	Problem-solving readiness score
Language & Literacy	Vocabulary production rate	Audio transcription AI	Early literacy trajectory band
Social-Emotional	Peer interaction frequency	Video observation coding	Regulation maturity index
Physical-Motor	Fine/gross motor precision	Wearable sensor data	Motor development pathway
Creative Expression	Divergent play episodes	Portfolio analysis	Creative capacity projection

### The AI-Responsive curriculum design (ARCD) framework

Synthesizing the three emergent constructs, this study proposes the *AI-Responsive Curriculum Design (ARCD) Framework* as an original theoretical contribution to the fields of early childhood education, educational technology, and curriculum studies. The ARCD Framework comprises three integrated principles:

**Principle 1 - Developmental Transparency:** AI systems integrated into ECE curriculum design must be designed to make their algorithmic reasoning legible and interrogable to educators. Trajectory predictions must be presented with confidence intervals, indicator variables, and plain-language explanations that enable educators to engage critically rather than passively with algorithmic output.

**Principle 2 - Ecological Sensitivity:** Following Bronfenbrenner’s (1979) ecological imperative, AI-responsive curriculum frameworks must incorporate multi-layer contextual data including family language environment, community socioeconomic context, and cultural developmental values as co-determinants of trajectory prediction, rather than treating individual behavioral interaction data as the sole input variable.

**Principle 3 - Professional Amplification:** AI tools must be explicitly designed to expand rather than replace educator professional judgment. This requires built-in mechanisms for educator feedback to the system (enabling continuous model refinement based on professional contextual knowledge), transparent documentation of algorithmic limitations, and professional development support that cultivates educators’ AI literacy without eroding their pedagogical confidence.

The ARCD Framework advances existing AIED scholarship ( Holmes et al., 2022; Luckin et al., 2023) by providing the first empirically grounded, phenomenologically derived framework specifically designed for early childhood curriculum contexts. It responds directly to Véliz et al.’s (2024) call for qualitative inquiry into educator experiences of AIED integration, and it extends Fleer’s (2021) critique of cognitive reductionism in ECE technology by providing a constructive alternative framework that honors the full developmental complexity of early childhood.

### Limitations and future research directions

This study carries several important limitations that circumscribe the transferability of its findings. First, the single-site design while providing depth and contextual richness limits the diversity of institutional and socioeconomic contexts represented in the data. The nine participants (six educators, two curriculum specialists, and one AI developer) at a single Makassar school constitute a purposively rich but inherently bounded dataset; findings therefore represent theoretical propositions and emergent constructs rather than generalizable claims. Future research should employ multi-site designs across urban, semi-urban, and rural ECE settings to capture the full range of ecological conditions within which AI-responsive curricula must operate. Validation through Delphi studies or expert panel review involving 15–20 international ECE and AI specialists would substantially strengthen the ARCD Framework before any claim of broader transferability is made. Second, the twelve-week fieldwork period, while sufficient for the emergence of robust phenomenological constructs, does not enable the tracking of long-term developmental trajectory outcomes for children in AI-responsive classrooms a critical gap that should be addressed through longitudinal mixed-methods studies incorporating standardized developmental assessments.

Third, the current study does not address the technical architecture of the deep learning models employed in the AI platform, relying on the AI Developer-Collaborator’s expert account rather than independent technical analysis. Future interdisciplinary research should bring together ECE scholars, learning scientists, and AI engineers to develop and validate deep learning model architectures specifically calibrated for early childhood developmental complexity. Fourth, the equity concerns surfaced in this study particularly the systematic underrepresentation of bilingual and indigenous developmental trajectories in current AI training datasets require urgent methodological attention, including the development of participatory AI design processes that actively involve indigenous communities and bilingual educators in defining developmental benchmarks.

## Conclusion

This study set out to investigate how AI-responsive frameworks can be conceptualized to support individualized developmental learning trajectories in preschool education, centering the phenomenological experiences of ECE educators and curriculum specialists at a single Indonesian preschool as the primary epistemic lens. The findings demonstrate that effective AI integration in early childhood curriculum design is not primarily a technical challenge but a *relational and pedagogical* one: it requires the deliberate construction of productive dialogue between algorithmic intelligence and educator professional wisdom.

The three emergent constructs Predictive Developmental Tracking, AI-Responsive Curriculum Adaptation, and Deep Learning as Pedagogical Scaffold together constitute the *AI-Responsive Curriculum Design (ARCD) Framework*: an original theoretical contribution that advances the field by providing the first phenomenologically grounded, early-childhood-specific framework for ethical and effective AI curriculum integration. The ARCD Framework’s three principles Developmental Transparency, Ecological Sensitivity, and Professional Amplification offer curriculum designers, educational technologists, and policy makers a theoretically robust and practically actionable guide for designing AI-responsive preschool curricula that are simultaneously intelligent, equitable, and humanizing.

Most critically, this study insists that the future of AI in early childhood education must be grounded in a foundational commitment: that technology serves the child, not the algorithm. Deep learning frameworks hold extraordinary promise for expanding the developmental intelligence available to educators and enabling truly personalized learning trajectories. However, this promise can only be realized if AI systems are designed with the full ecological, cultural, and relational complexity of early childhood development at their center and if educators are empowered, not displaced, as the ultimate architects of developmentally responsive, equity-affirming preschool education.

## Ethical considerations

Ethical approval for this study was obtained from the Research Ethics Committee of Universitas Negeri Makassar, Indonesia, under Ethical Approval Protocol No. 2024-ECE-AI-07. The study complied with institutional ethical standards for research involving human participants and was conducted in accordance with the principles of the Declaration of Helsinki. All participants were informed about the objectives of the study, the voluntary nature of participation, anonymity, and the confidentiality of their responses. Written and verbal informed consent were obtained from all participants prior to data collection. Verbal informed consent was used as an additional measure to ensure that participants fully understood the study procedures and their rights before participating. Children observed during classroom activities were not recruited as research participants, and no personally identifiable information about children was collected.

## Data Availability

Zenodo: Interview Instrument and Transcript for Research Entitled Designing an AI-Responsive Early Childhood Curriculum: A Conceptual Framework for Supporting Developmental Learning Trajectories in Preschool Education DOI 10.5281/zenodo.20839909 ( Hasan et al., 2026). This project contains the following extended data:
1. Interview Instrument and Transcript
