# Designing an AI-Responsive Early Childhood Curriculum: A Conceptual Framework for Supporting Developmental Learning Trajectories in Preschool Education

**DOI:** 10.12688/f1000research.185645.2

**Published:** 2026-10-06

**Authors:** Herlina Herlina, Chandra Apriyansyah, Rusmayadi Rusmayadi, Herman Herman

**Affiliations:** 1 Early Childhood Education Program, Universitas Negeri Makassar, Makassar, South Sulawesi, 90222, Indonesia; 2 Doctoral Program in Early Childhood Education, Universitas Negeri Jakarta, East Jakarta, DKI Jakarta, 13320, Indonesia

**Keywords:** Artificial Intelligence; Early Childhood Education; Curriculum Design; Deep Learning; Teacher Agency; AI-Responsive Curriculum Design (ARCD)

## Abstract

**Background:**

The rapid integration of artificial intelligence (AI) and deep learning technologies into education creates new opportunities for personalized learning in early childhood education (ECE). Yet limited research has examined how educators interpret and enact AI-responsive curriculum decisions in preschool settings, particularly in culturally diverse contexts.

**Purpose:**

This study explores educators’ experiences in designing and implementing AI-responsive curricula and develops a conceptual framework explaining how AI can inform curriculum decision-making for young children without displacing teacher judgment.

**Method:**

An interpretive phenomenological study was conducted at TK Harapan Bangsa, Makassar, Indonesia, with nine adult participants: six preschool teachers, two curriculum specialists, and one AI tool developer-collaborator whose expert interview supplied technical context. Data comprised two in-depth interviews with each teacher, interviews with curriculum specialists and the developer-collaborator, 18 non-participant observations, and analysis of AI-supported planning documents. MAXQDA 2024 supported data management. Giorgi’s phenomenological method was operationalized through first-order meaning units, second-order interpretive themes, and third-order composite constructs. Triangulation, reflexive memoing, an audit trail, member checking, and peer debriefing supported trustworthiness.

**Findings:**

Three interconnected constructs emerged. Predictive Developmental Tracking describes AI-supported identification of developmental readiness and possible concerns. AI-Responsive Curriculum Adaptation captures teachers’ active interrogation and contextual modification of algorithmic recommendations. Deep Learning as Pedagogical Scaffold positions AI as a support that amplifies, rather than replaces, professional expertise. Together, the constructs informed the AI-Responsive Curriculum Design (ARCD) Framework.

**Conclusion:**

The ARCD Framework explains AI-responsive curriculum implementation as a relational process combining predictive insight, ecological sensitivity, and educator agency. It offers implications for curriculum policy, teacher professional development, and ethical AI integration in ECE.

## Introduction

The twenty-first century has ushered in an unprecedented convergence of educational science and artificial intelligence (AI), redefining what is possible within early childhood education (ECE). The earliest years of learning, spanning birth to six years, represent a highly formative period of human development ( Shonkoff & Phillips, 2000). Concurrently, machine learning, deep learning, adaptive systems, and generative AI have intensified scholarly and practitioner interest in whether computational tools can meaningfully support individualized developmental pathways for young learners ( Holmes et al., 2019; Kasneci et al., 2023; Yan et al., 2024).

Two scholarly trajectories have often developed in relative isolation. The first concerns AI applications in formal schooling, especially performance prediction, learning analytics, adaptive content, and generative support. The second concerns developmentally appropriate ECE, which emphasizes play-based learning, holistic development, relational pedagogy, and educator judgment ( NAEYC, 2020; Fleer, 2021). Their intersection – the design of AI systems responsive to the fluid, multimodal, cultural, and emergent character of early childhood development – remains empirically limited. Chen’s (2024) scoping study identified only 18 empirical journal articles across 11 countries through 2023 and called for more research involving educators, families, policymakers, ethical questions, and sociocultural comparisons.

The gap is consequential. When AI systems are used with young children, benefits depend on adult mediation, developmental appropriateness, trustworthy outputs, and protection from privacy, bias, and over-reliance risks. Recent studies illustrate both promise and caution. An eight-week intervention found that an intentionally designed program could support young children’s AI literacy ( Su & Yang, 2024a), while a comparison of storytelling with ChatGPT and parents showed different interaction patterns and reinforced the continuing importance of human relational engagement ( Chin et al., 2024). A recent systematic review similarly concluded that generative AI in ECE is best positioned as a complement to adult guidance, not a replacement for it ( Zhang et al., 2026).

The Indonesian preschool context provides a particularly instructive case. Indonesia’s national preschool curriculum emphasizes holistic child development across cognitive, language, social-emotional, physical-motor, and value-moral domains ( Kemendikbud, 2022). At the same time, digitalization initiatives create implementation tensions: educators are expected to use digital and AI-supported tools while navigating pronounced developmental, linguistic, and cultural heterogeneity.

Against this background, this study investigates how AI-responsive curricula can be conceptualized and implemented to support individualized developmental learning trajectories in preschool education. The inquiry prioritizes educators’ lived experiences, professional judgment, and interpretive meaning-making. Rather than evaluating algorithmic accuracy or child outcomes, the study asks how educators negotiate AI-supported information within the relational and developmental complexity of one Indonesian preschool.

The study is anchored in three complementary frameworks. Vygotsky’s Zone of Proximal Development (ZPD) ( Vygotsky, 1978) provides a basis for considering AI as a possible scaffold for developmental readiness. Bronfenbrenner’s Ecological Systems Theory (1979) situates development within interacting family, school, community, and cultural systems. Universal Design for Learning (UDL) ( CAST, 2018) emphasizes flexible and equitable curriculum pathways responsive to learner variability. Recent human-centered guidance also stresses that teachers require AI competencies encompassing ethics, AI pedagogy, professional learning, and protection of human agency ( Miao & Cukurova, 2024).

This research addresses two questions: (1) How do ECE educators conceptualize and negotiate the integration of AI-responsive systems into preschool curriculum design? and (2) What emergent principles characterize an effective AI-Responsive Curriculum Design (ARCD) framework for supporting developmental learning trajectories in early childhood settings?

## Literature review

### Artificial intelligence and deep learning in educational contexts

AI in education includes predictive systems that forecast performance, adaptive systems that adjust learning content, and generative systems that create or transform materials ( Holmes et al., 2019). Deep learning architectures can process complex sequential and multimodal data, while large language and foundation models enable flexible content generation and dialogue ( LeCun et al., 2015; Goodfellow et al., 2016; Brown et al., 2020; Bommasani et al., 2021; Kasneci et al., 2023). Explainable AI is especially important in education because educators need to interrogate the basis of algorithmic suggestions rather than treat opaque outputs as authoritative ( Arrieta et al., 2020; Khosravi et al., 2022).

Recent evidence underscores the importance of educator agency. Kindergarten teachers have described ChatGPT as simultaneously useful and limited, identifying potential support for lesson planning and pedagogical knowledge alongside concerns about accuracy, resources, policy, and training ( Su & Yang, 2024b). In human learning more broadly, generative AI can support personalization, feedback, and new forms of assessment, but model imperfection and over-reliance require critical AI literacy and pedagogical oversight ( Yan et al., 2024). UNESCO’s teacher framework therefore places a human-centered mindset, ethics, AI foundations, AI pedagogy, and professional learning at the center of responsible adoption ( Miao & Cukurova, 2024).

### Early childhood development and the limitations of current AI tools

Early childhood development is domain-interdependent, non-linear, and profoundly influenced by relational and environmental contexts ( Shonkoff & Phillips, 2000). Cognitive, language-literacy, social-emotional, physical-motor, and value-moral development do not occur in isolation. This systemic quality challenges tools built around narrow or reductive performance indicators.

Chen’s (2024) review found four broad ECE affordances: AI as an interactive learning or information tool, a technology for predicting or classifying conditions, an adaptive means of personalizing learning, and a subject that children learn about. Yet the evidence base was geographically uneven and called for closer examination of sociocultural context, equity, and stakeholder perspectives. Zhang et al. (2026) likewise found that benefits reported across 29 generative-AI studies were consistently dependent on active adult mediation, while technical limitations, content quality, and accuracy remained recurring concerns.

Empirical work with young children also cautions against assuming that AI interactions reproduce human relational qualities. Chin et al. (2024), working with eight child-parent dyads, found that children aged five to six engaged differently with ChatGPT and parents during creative storytelling. Such findings support a design principle in which AI augments adult-child interaction and teacher decision-making rather than substituting for them.

### Vygotsky’s zone of proximal development and AI scaffolding

Vygotsky’s (1978) ZPD describes the space between what a child can accomplish independently and what becomes possible with knowledgeable support. Traditionally, support comes from a teacher, parent, or peer. AI can be conceptualized as a digital scaffold only when its support remains contingent, adjustable, and subordinate to pedagogical goals. The classic scaffold characteristics of contingency, fading, and intentionality ( Wood et al., 1976; Palincsar & Brown, 1984) imply that AI recommendations should respond to evidence of readiness without becoming permanent or directive.

### Bronfenbrenner’s ecological systems theory and culturally situated AI

Bronfenbrenner’s (1979) Ecological Systems Theory situates development within interacting microsystem, mesosystem, exosystem, and macrosystem influences. Applied to AI-responsive curriculum design, the framework suggests that classroom interaction data alone cannot fully explain a child’s developmental trajectory. Family language practices, school-family relationships, community resources, and cultural expectations also matter.

This ecological imperative aligns with recent ECE teacher-education research. Li and Yu (2025) found that culturally responsive AI preparation can strengthen preservice teachers’ AI literacy while revealing the need to evaluate bias, representation, and cultural fit. Such work reinforces the requirement that educators critically adapt AI-generated materials rather than accept them at face value.

### Universal design for learning and equitable AI curriculum design

UDL calls for multiple means of representation, action and expression, and engagement ( CAST, 2018). Within AI-responsive ECE, learner variability should be treated as an expected feature of development, not a deficit. Equity-centered UDL also requires attention to whether algorithms misclassify culturally or linguistically diverse children and whether teachers can understand, challenge, and override recommendations ( Waitoller & King Thorius, 2016).

### Gaps in the literature and research rationale

Three gaps motivate this study. First, recent reviews continue to identify limited stakeholder-centered qualitative inquiry in ECE, particularly research on educators’ lived negotiations with AI ( Chen, 2024; Zhang et al., 2026). Second, curriculum frameworks often travel from older educational levels without sufficient attention to the ecological, play-based, and relational qualities of early childhood. Third, existing studies demonstrate both opportunities and limitations, but offer limited empirically grounded explanation of how teachers combine algorithmic recommendations with contextual judgment. This study addresses these gaps through a phenomenological analysis of AI-responsive curriculum work in one Indonesian preschool.

## Methodology

### Research design and paradigm

This study adopted a qualitative phenomenological design grounded in the interpretivist paradigm. The hermeneutic tradition associated with Heidegger (1962), Gadamer (1975), and van Manen (2016) was appropriate because the research questions concern how educators experience, interpret, and assign meaning to AI integration in ECE. The study did not test algorithmic performance or compare AI-assisted and non-AI-assisted child outcomes. Its purpose was to construct a theoretically informed account of educators’ pedagogical negotiations.

### Research setting

The research was conducted at TK Harapan Bangsa, a public preschool in Tamalanrea, Makassar, South Sulawesi, Indonesia. The setting was purposively selected because it served a socioeconomically diverse urban population, had participated in a digital-integration pilot since 2023, and supported reflective teacher professional development. These characteristics provided an information-rich context for examining AI-supported curriculum planning.

### Participants

Purposive maximum-variation sampling ( Patton, 2015) was used to include differences in professional experience, developmental-domain expertise, and prior AI engagement. The study included nine adult participants:

1. Six ECE classroom educators with two to fifteen years of preschool experience. All held at least a Diploma IV or bachelor’s degree in ECE. These educators were the primary phenomenological participants because they directly experienced AI-supported planning and classroom decision-making.
2. Two curriculum specialists affiliated with Dinas Pendidikan Kota Makassar. They contributed institutional and curriculum-development perspectives.
3. One AI tool developer-collaborator. This participant completed a single extended expert interview and served as an additional technical informant; the interview contextualized system design and did not replace educator accounts as the principal experiential evidence.

Written and verbal informed consent were obtained from all adult participants. Participation was voluntary, withdrawal was permitted without consequence, and coded identifiers were used. Ethical clearance was granted by the Research Ethics Committee of Universitas Negeri Makassar (Protocol No. 2024-ECE-AI-07).

**Table 1.** Demographic profile of study participants (n = 9).

Characteristic	Category	Frequency, n (%)
Gender	Male	6 (67%)
Gender	Female	3 (33%)
Participant role	ECE classroom educator	6 (67%)
Participant role	Curriculum specialist	2 (22%)
Participant role	AI tool developer-collaborator	1 (11%)
Professional experience	1–5 years	3 (33%)
Professional experience	6–10 years	3 (33%)
Professional experience	>10 years	3 (33%)
AI integration experience	Novice (<1 year)	4 (44%)
AI integration experience	Intermediate (1–2 years)	3 (33%)
AI integration experience	Experienced (>2 years)	2 (22%)

### Data Collection Procedures

Data were collected during twelve weeks through three complementary sources:

1. Semi-structured phenomenological interviews. Each of the six classroom educators completed two in-depth interviews averaging 65 minutes. Questions addressed initial encounters with AI tools, moment-to-moment negotiation of algorithmic suggestions, and interpretations of developmental-trajectory information. The curriculum specialists participated in curriculum-focused interviews, and the developer-collaborator completed one extended expert interview for technical context. Interviews were conducted in Bahasa Indonesia, audio-recorded with consent, and transcribed verbatim.
2. Non-participant classroom observations. Eighteen 45-minute sessions were distributed across the six educator participants. A structured protocol and field notes documented occasions when educators consulted trajectory information, differentiated activities, or departed from AI recommendations. Observations focused on educator-AI decision sequences; children were not recruited as research participants.
3. Document analysis. The corpus included AI-generated weekly learning-trajectory reports, educator reflective journals, lesson-plan adaptations, and parent-communication records. Documents were examined in relation to purpose, audience, and institutional context following Bowen (2009).

The three sources enabled methodological triangulation ( Denzin, 2012). No personally identifying child information was collected for research reporting. References to classroom situations were de-identified, and the conceptual framework was developed from adult participant accounts, observations of professional practice, and planning documents.

### Data analysis

Analysis followed Giorgi’s (2009) systematic phenomenological method, adapted to educational research. MAXQDA 2024 supported data organization, retrieval, memoing, and comparison; it did not automatically code data or generate themes. The analysis proceeded through five iterative operations:

1. Familiarization and horizonalization. Transcripts, observation notes, and documentary excerpts were read repeatedly. Statements relevant to the experience of AI-responsive curriculum work were initially treated as horizons of equal significance.
2. First-order meaning units. Relevant passages were segmented and assigned concise participant-near labels that preserved the action or meaning expressed in the source. In-vivo wording was retained where it clarified the participant’s perspective. For example, P4’s statement “That’s me using the algorithm” was coded as teacher ownership of contextual adaptation.
3. Second-order interpretive themes. Related meaning units were compared within and across source types. The research team examined convergence, elaboration, tension, and disconfirming evidence. Codes were grouped into themes such as early signal detection, equity-oriented bias recognition, contextual interrogation, professional synthesis, and complementary forms of knowing.
4. Imaginative variation and third-order composite constructs. The team considered which features remained essential when contextual conditions were varied. Second-order themes were then organized into three composite constructs: Predictive Developmental Tracking, AI-Responsive Curriculum Adaptation, and Deep Learning as Pedagogical Scaffold.
5. Textural-structural synthesis. Descriptions of what participants experienced were integrated with descriptions of how they experienced it. The final ARCD Framework was developed from this synthesis and checked against supporting evidence from interviews, observations, and documents.

The labels first-order, second-order, and third-order are used here to make levels of abstraction visible to readers; they do not replace Giorgi’s phenomenological logic with a grounded-theory procedure. The MAXQDA code system grouped sources by participant role and data type and stored code definitions, inclusion and exclusion criteria, exemplar excerpts, and linked memos. The audit trail contained dated reflexive memos, successive codebook versions, decisions to merge, split, or rename codes, links between constructs and supporting excerpts, and records of changes arising from member checking and peer debriefing.

### Researcher reflexivity, trustworthiness, and positionality

Because interpretation is co-constructed within an interpretivist study, the researchers treated their professional commitments to ECE, curriculum innovation, and educational technology as potential sources of both insight and bias. Before and during coding, reflexive memos recorded expectations about AI’s possible benefits and risks, reactions to participant accounts, and reasons for interpretive choices. Analytic discussions deliberately retained reservations, algorithmic-bias examples, and occasions when teachers rejected or reshaped AI recommendations, rather than privileging only favorable accounts. This process supported awareness of how researchers’ positions influenced the developing interpretation.

Trustworthiness was strengthened through triangulation across interviews, observations, and documents; an explicit audit trail; and attention to discrepant evidence. At the end of analysis, summaries of the emergent constructs and evidence were shared with four of the six educator participants, who confirmed that the interpretation resonated with their experiences. Two senior colleagues external to the study conducted peer debriefing to challenge assumptions and alternative explanations.

## Findings and discussion

The analysis generated three third-order constructs representing distinct but interdependent dimensions of participants’ experience. Table 2 makes the analytical progression visible; the sections below provide fuller phenomenological and theoretical interpretation.

**Table 2.** Coding matrix: from data excerpt to first-order meaning unit, second-order theme, and third-order construct.

Illustrative data excerpt	First-order meaning unit	Second-order interpretive theme	Third-order composite construct
“The AI system flagged a child’s reduced fine-motor engagement before the teacher noticed any change.” (Field note, Week 3)	AI flag precedes teacher recognition	Early signal detection and retrospective confirmation	Predictive Developmental Tracking
“When I reviewed my observation notes... I could see what the algorithm had detected... It’s sharpening [my observation].” (P1, Interview 2, Week 9)	Teacher recalibrates judgment after checking own notes	Amplified perception through data-observation comparison	Predictive Developmental Tracking
“The AI’s literacy indicators were calibrated exclusively to Bahasa Indonesia benchmarks... The AI became a mirror [of] our curriculum’s cultural assumptions.” (CS1, Interview 1, Week 4)	System output exposes language-benchmark bias	Equity-centered diagnosis and variable redesign	Predictive Developmental Tracking
“The curriculum module shifted from visual-spatial activities to language-rich tasks after detecting a plateau.” (Observation log, Week 5)	Content sequence changes after a detected plateau	Algorithmic reception and adaptive sequencing	AI-Responsive Curriculum Adaptation
“The algorithm doesn’t know [the family distress]... I introduced sensory play... That’s me using the algorithm.” (P4, Interview 2, Week 7)	Context overrides or reshapes algorithmic advice	Contextual interrogation and professional synthesis	AI-Responsive Curriculum Adaptation
“A 73% probability... should be read as an invitation to investigate, not an instruction to execute.” (D1, expert interview, Week 6)	Probabilities invite inquiry rather than compliance	Transparent and non-deterministic recommendation	AI-Responsive Curriculum Adaptation
“The AI-generated trajectory report helped [the educator] discuss development with parents using more specific evidence.” (Interview record, Week 8)	Trajectory evidence strengthens parent communication	AI-mediated evidence for professional practice	Deep Learning as Pedagogical Scaffold
“The AI knows patterns... I know my children... together [they are] more powerful than either one alone.” (P2, Interview 2, Week 11)	Algorithmic and relational knowing are complementary	Professional amplification and retained teacher authority	Deep Learning as Pedagogical Scaffold

Note: The matrix is a concise extract of the complete audit trail. “Equity-centered diagnosis” is treated as a second-order theme within Predictive Developmental Tracking, not as a fourth third-order construct.

**Figure 1.**
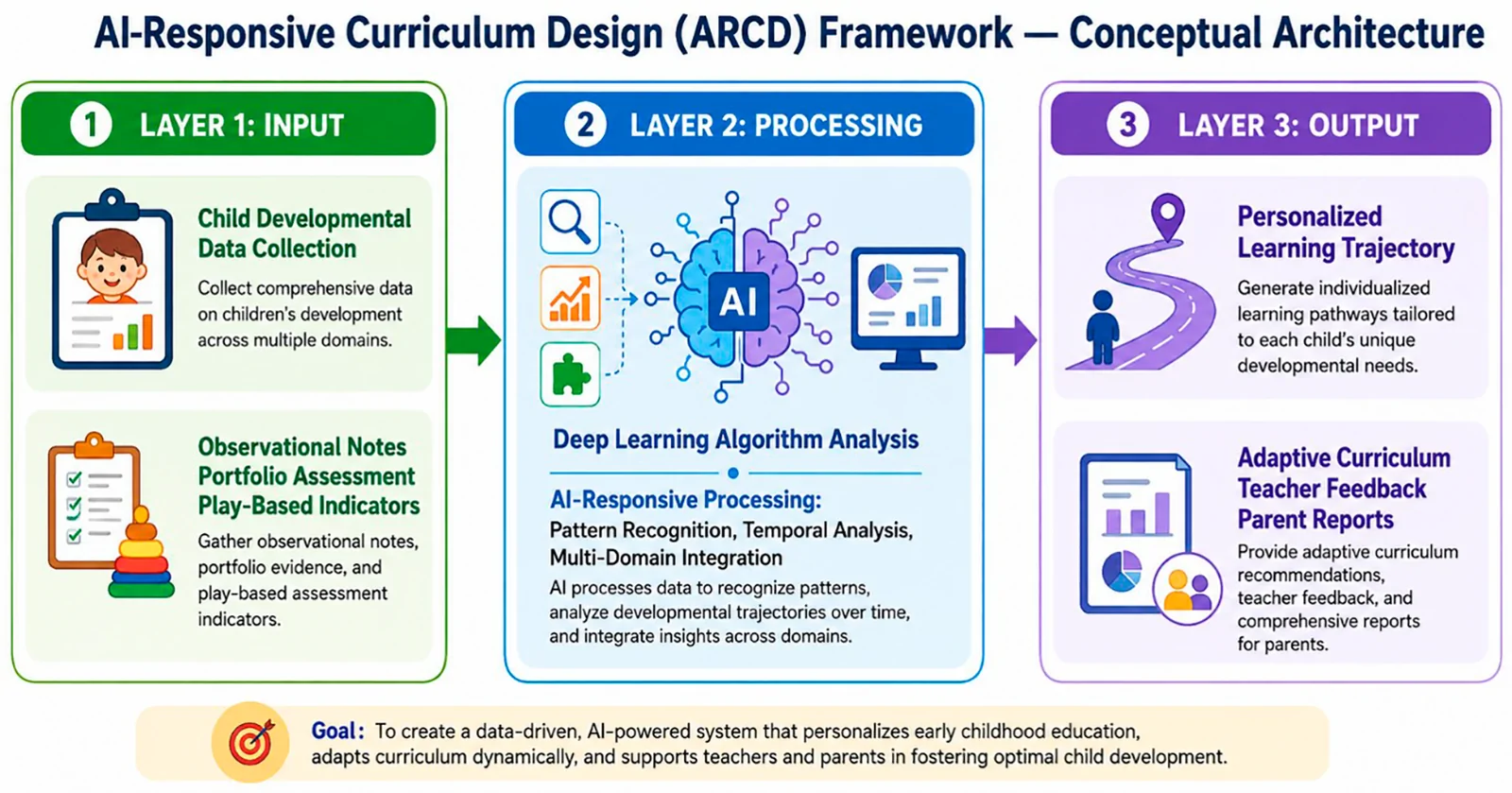
AI-Responsive Curriculum Design (ARCD) Framework – conceptual architecture.

### Construct 1: Predictive Developmental Tracking: AI as Early Warning System

Predictive Developmental Tracking describes participants’ experience of AI-supported trajectory information surfacing a developmental concern, pattern, or readiness indicator before it became obvious through routine classroom observation. Educators often described initial dissonance followed by professional recalibration: algorithmic information disrupted an assumption, prompted re-examination of observational evidence, and sometimes sharpened subsequent attention.

*“When the AI flagged that Rafi was showing micro-patterns of reduced engagement in spatial reasoning tasks - specifically a 23% decline in interaction duration across three consecutive sessions - I initially dismissed it. I thought, ‘I know this child; he’s fine.’ But when I went back and reviewed my own observation notes from those same sessions, I could see in retrospect what the algorithm had detected in real time. That moment changed how I relate to the data. It’s not replacing my observation; it’s sharpening it.”* (Educator P1, Interview 2, Week 9).

This account illustrates amplified perception, not automated diagnosis. The teacher checked the AI flag against personal observation notes and retained interpretive responsibility. In Vygotskian terms, the system extended the evidence available for professional reasoning, but did not become the final authority over the child’s development.

The curriculum specialists added an institutional and equity dimension:

*“The system generated a report showing that children from bilingual Bugis-Indonesian households were systematically showing delayed trajectory predictions in early literacy, not because they were cognitively behind, but because the AI’s literacy indicators were calibrated exclusively to Bahasa Indonesia benchmarks. We used that finding to redesign the tracking variables. The AI became a mirror that showed us our own curriculum’s cultural assumptions.”* (Curriculum Specialist CS1, Interview 1, Week 4).

Here, predictive tracking exposed a limitation in the benchmark itself. Equity-centered diagnosis therefore operates as a theme within Predictive Developmental Tracking: the value of the system lies partly in making cultural assumptions visible and revisable. This interpretation accords with recent calls to examine sociocultural influences and the AI divide in ECE research ( Chen, 2024) and with culturally responsive teacher preparation for AI use ( Li & Yu, 2025).

### Construct 2: AI-Responsive curriculum adaptation – Negotiating algorithm and pedagogy

AI-Responsive Curriculum Adaptation describes how educators moved from receiving a recommendation to interrogating it and then producing a contextually appropriate pedagogical response. Participants did not describe implementation as passive acceptance. Instead, they combined algorithmic reception, contextual interrogation, and professional synthesis.

*“The AI told me Sari needed more fine-motor challenges - cutting, threading, building. But I knew that last week her parents had separated, and she was coming to school visibly distressed. The algorithm doesn’t know that. So I took the AI’s developmental logic and applied it through an emotional lens - I introduced sensory play activities that built fine-motor capacity while also providing calming, self-regulatory experiences. That’s not the algorithm; that’s me using the algorithm.”* (Educator P4, Interview 2, Week 7).

The quote shows that adaptation depended on ecological knowledge unavailable in the algorithmic output. The teacher retained authority over how a developmental objective should be enacted. This form of professional mediation is consistent with recent evidence that ECE teachers value generative AI for planning support while remaining concerned about accuracy, contextual fit, training, and policy support ( Su & Yang, 2024b).

The developer-collaborator described the intended role of probability displays:

*“We deliberately designed the system to present trajectory predictions as probability ranges rather than definitive assessments precisely because we knew that the contextual variables educators carry in their heads are irreducibly important. A 73% probability that a child will benefit from increased spatial-reasoning challenges should be read as an invitation to investigate, not an instruction to execute.”* (AI Developer D1, Expert Interview, Week 6).

This account supports a principle of non-deterministic and explainable recommendation. It also aligns with UDL: AI output should expand the repertoire of possible responses while teachers choose developmentally and culturally appropriate forms of engagement.

### Construct 3: Deep learning as pedagogical scaffold – AI Amplifying teacher expertise

Deep Learning as Pedagogical Scaffold represents participants’ longer-term reorientation from apprehension toward conditional professional amplification. Four of the six teachers initially worried that AI might threaten autonomy. By later interviews, participants more often described a division of epistemic labor: the system identified patterns across data, while teachers supplied relational, contextual, emotional, and cultural knowledge.

*“In the beginning, I thought, ‘This machine is going to tell me how to teach.’ But over the weeks, I began to see it differently. The AI doesn’t know my children; it knows patterns in their data. I know my children. What happens when you put those two kinds of knowing together is something more powerful than either one alone. It’s like having a very data-literate teaching assistant who never forgets anything and who reminds you to look at things you’ve unconsciously stopped noticing.”* (Educator P2, Interview 2, Week 11).

The scaffold was conditional. Participants emphasized transparency, probabilistic rather than definitive output, cultural and linguistic sensitivity, and the teacher’s ability to question or reject a recommendation. These conditions echo human-centered AI competence and recent reviews concluding that AI in ECE should complement active adult guidance ( Miao & Cukurova, 2024; Zhang et al., 2026).

To illustrate how the ARCD principles may be operationalized across developmental domains, Table 3 maps selected domains to illustrative AI indicators, potential data sources, and possible trajectory outputs. These examples are conceptual and are not validated diagnostic measures.

**Table 3.** Illustrative developmental-domain mapping for the proposed ARCD Framework.

Developmental domain	Illustrative AI indicator	Potential data source	Possible trajectory output
Cognitive	Pattern-recognition engagement	Digital interaction logs	Problem-solving readiness indicator
Language and literacy	Vocabulary-production pattern	Teacher-verified transcription	Early-literacy trajectory band
Social-emotional	Peer-interaction pattern	Structured observation coding	Regulation-support indicator
Physical-motor	Fine/gross-motor participation	Teacher observation or consented sensor data	Motor-development support pathway
Creative expression	Divergent-play episodes	Portfolio analysis	Creative-capacity profile

### The AI-Responsive curriculum design (ARCD) framework

Synthesizing the three constructs, the ARCD Framework comprises three integrated principles:

1. Developmental Transparency. AI-supported predictions should be legible and open to interrogation. Educators need indicator definitions, uncertainty information, and plain-language explanations.
2. Ecological Sensitivity. Family language, classroom relationships, community conditions, and cultural developmental values should inform interpretation. Individual interaction traces should not be treated as self-sufficient evidence.
3. Professional Amplification. AI should extend, not replace, educator judgment. Systems should support teacher feedback, documentation of limitations, and professional development in critical AI literacy.

The framework contributes an empirically grounded explanation of AI-responsive ECE curriculum work. It connects predictive information, teacher adaptation, and professional agency while incorporating recent evidence that adult mediation, cultural responsiveness, and AI literacy are central to responsible use ( Chen, 2024; Li & Yu, 2025; Zhang et al., 2026).

### Limitations and future research directions

The single-site design and nine adult participants provide depth but limit transferability across institutional, socioeconomic, cultural, and technological contexts. Findings are theoretical propositions grounded in this setting, not statistically generalizable claims. Multi-site studies across urban, semi-urban, rural, and linguistically diverse ECE settings should examine which ARCD elements transfer and which require local redesign.

The twelve-week fieldwork period does not establish long-term developmental outcomes for children. Longitudinal mixed-methods research would be required to examine whether AI-responsive curriculum decisions contribute to sustained benefits and whether risks such as over-reliance, misclassification, or reduced relational interaction emerge over time.

The study did not independently validate the technical architecture, accuracy, fairness, or security of the AI platform. Future interdisciplinary research should include ECE scholars, learning scientists, AI engineers, families, and ethics specialists. Participatory design is especially important for defining culturally appropriate benchmarks and ensuring that bilingual, indigenous, and marginalized communities shape the variables used to represent development.

Finally, the ARCD Framework requires prospective validation. Delphi review, design-based research, and carefully governed pilot studies could examine clarity, usability, ethical adequacy, and contextual fit before broader implementation.

## Conclusion

This study examined how educators conceptualized and negotiated AI-responsive curriculum work in one Indonesian preschool. The findings suggest that responsible AI integration is primarily a relational and pedagogical process: algorithmic pattern recognition becomes educationally meaningful only through contextual interpretation, ethical judgment, and developmentally appropriate action by educators.

Predictive Developmental Tracking, AI-Responsive Curriculum Adaptation, and Deep Learning as Pedagogical Scaffold form the ARCD Framework. Its principles of Developmental Transparency, Ecological Sensitivity, and Professional Amplification offer an empirically grounded, early-childhood-specific guide for curriculum design, teacher professional development, and policy. The framework does not treat AI as an autonomous decision-maker. It positions AI as a contestable source of evidence within a human-led curriculum process.

The central commitment is that technology should serve children’s holistic development and strengthen relationships rather than subordinate education to algorithmic output. Realizing that commitment requires transparent systems, culturally responsive data practices, critical AI literacy, and educators who remain the final interpreters of what developmentally appropriate curriculum action should be.

## Ethical considerations

Ethical approval was obtained from the Research Ethics Committee of Universitas Negeri Makassar, Indonesia (Protocol No. 2024-ECE-AI-07). Adult participants received information about the study’s objectives, procedures, voluntary nature, confidentiality, and right to withdraw. Written and verbal informed consent were obtained before data collection. Children present during routine classroom observations were not recruited as research participants, and no personally identifying child information is reported.

## Data Availability

Zenodo: Interview Instrument and Transcript for Research Entitled Designing an AI-Responsive Early Childhood Curriculum: A Conceptual Framework for Supporting Developmental Learning Trajectories in Preschool Education. DOI 10.5281/zenodo.20839909 ( Hasan et al., 2026). This project contains the following extended data:
1. Interview Instrument and Transcript
