# Supplementary material for: Designing an AI-Responsive Early Childhood Curriculum: A Conceptual Framework for Supporting Developmental Learning Trajectories in Preschool Education
Source: F1000Res. 2026 Oct 6;15:1172. Originally published 2026 Jul 16. [Version 2] doi: 10.12688/f1000research.185645.2 (PMC13673081; doi:10.12688/f1000research.185645.2)
Supplement: Peer review history

# Reviewer report: Reviewer response for version 2

**DOI:** 10.5256/f1000research.209452.r527773

**Published:** 2026-10-27

**Authors:** Irma Yuliantina

**Affiliations:** 1 Indonesian Coalition for Holistic-Integrative Early Childhood Development, Jakarta, Jakarta, Indonesia; 2 Universitas Panca Sakti Bekasi, Bekasi, West Java, Indonesia

The authors have adequately addressed the comments and suggestions provided in the previous review. The revised manuscript demonstrates improvements in clarity, presentation, and overall quality. I appreciate the authors’ efforts and responsiveness in incorporating the feedback. I have no further comments or additional recommendations at this stage.

Is the work clearly and accurately presented and does it cite the current literature?

Yes

If applicable, is the statistical analysis and its interpretation appropriate?

Yes

Are all the source data underlying the results available to ensure full reproducibility?

Yes

Is the study design appropriate and is the work technically sound?

Yes

Are the conclusions drawn adequately supported by the results?

Yes

Are sufficient details of methods and analysis provided to allow replication by others?

Yes

Reviewer Expertise:

Early Childhood EducationEarly Childhood Education CurriculumArtificial Intelligence in EducationCurriculum Design and DevelopmentAdaptive and Personalized Learning

I confirm that I have read this submission and believe that I have an appropriate level of expertise to confirm that it is of an acceptable scientific standard.

# Reviewer report: Reviewer response for version 2

**DOI:** 10.5256/f1000research.209452.r527774

**Published:** 2026-10-15

**Authors:** Diah Andika Sari

**Affiliations:** 1 Universitas Muhammadiyah Jakarta, Jakarta, Special Capital Region of Jakarta, Indonesia

The authors have adequately addressed this comment. The revisions improve the clarity and transparency of the data analysis process.

Is the work clearly and accurately presented and does it cite the current literature?

Yes

If applicable, is the statistical analysis and its interpretation appropriate?

Not applicable

Are all the source data underlying the results available to ensure full reproducibility?

Partly

Is the study design appropriate and is the work technically sound?

Yes

Are the conclusions drawn adequately supported by the results?

Yes

Are sufficient details of methods and analysis provided to allow replication by others?

Partly

Reviewer Expertise:

Early Childhood Education, The impact of gadgets on child development, ECE Management & Curriculum, Play & movement , Dance & Creative Movement

I confirm that I have read this submission and believe that I have an appropriate level of expertise to confirm that it is of an acceptable scientific standard.

# Reviewer report: Reviewer response for version 2

**DOI:** 10.5256/f1000research.209452.r528542

**Published:** 2026-10-13

**Authors:** Hardiyanti Pratiwi

**Affiliations:** 1 Universitas Negeri Yogyakarta, Yogyakarta, Special Region of Yogyakarta, Indonesia

Major Concerns and Requests for Clarification

 1. What form of AI integration was actually implemented in the ECE curriculum?

 The manuscript describes Predictive Developmental Tracking, AI-Responsive Curriculum Adaptation, and Deep Learning as Pedagogical Scaffold as the main components of the proposed framework. However, the Methods section (pp. 6–7) does not clearly identify the AI system used, its technical architecture, or how it generated developmental indicators and pedagogical recommendations. Figure 1 (p. 9) presents a data-processing architecture, while Table 3 (p. 11) lists potential data sources and indicators that are described as illustrative rather than validated measures. Could the authors clarify which AI system was actually used, how it operated, what data it processed, and which components of the proposed framework were implemented in practice rather than developed conceptually? Was AI used primarily to support lesson planning, or did the system actually analyse children's developmental data and generate predictive recommendations?

 2. How were AI-generated developmental predictions validated, and how was children's data protected?

 The Findings section (pp. 9–10) reports a 23% decline in interaction duration and a 73% probability that a child would benefit from additional spatial-reasoning activities. However, the manuscript does not explain how these figures were generated or whether the underlying predictions were validated. The authors also acknowledge that the AI platform's accuracy, fairness, and technical architecture were not independently evaluated (p. 11). Could the authors clarify whether these figures were generated by an operational AI system or are illustrative examples? If they were actual outputs, what model, variables, and validation procedures supported them? Furthermore, the Methods section (p. 7) states that no personally identifying child information was collected for research reporting but does not clarify whether individual children's data were entered into the AI system or processed by an external provider. What data were submitted to the AI platform, where were they processed and stored, and what measures ensured children's privacy and compliance with the relevant ethical requirements? De-identification in the manuscript does not necessarily establish that children's data were protected during AI processing.

 3. Did the participants actually design and implement an AI-responsive curriculum?

 The Participants section (p. 6) identifies six teachers as the primary phenomenological participants, supported by two curriculum specialists and one AI developer. The Data Collection Procedures section (p. 7) refers to AI-generated learning-trajectory reports, lesson-plan adaptations, and classroom observations. However, the manuscript does not clearly distinguish curriculum design from the use of AI to modify individual learning activities. Could the authors clarify whether the teachers were involved in designing the AI system and curriculum framework, or whether they primarily used an existing AI tool to adapt lesson plans and classroom activities? Who established the developmental indicators, curriculum objectives, and decision rules underlying the recommendations? What evidence demonstrates that a curriculum was actually designed and implemented, rather than those teachers used AI-assisted planning in their existing practice? The single-site design is not inherently problematic, but the scope of the claims must correspond to the practices documented in this setting.

 4. What precisely constitutes an AI-responsive curriculum, and what are its defining indicators?

 The Introduction and Research Questions (pp. 4–5) position AI-responsive curriculum design as the central focus of the study. However, the proposed principles of Developmental Transparency, Ecological Sensitivity, and Professional Amplification (pp. 11) largely concern transparency, contextual responsiveness, and teacher judgment, which are also relevant to conventional ECE curriculum design. Could the authors provide a clear operational definition of an AI-responsive curriculum and explain how it differs from existing approaches to adaptive or developmentally responsive curriculum planning? What specific indicators determine whether a curriculum is genuinely AI-responsive? How does AI-generated information trigger or inform curriculum changes, and how are these changes documented and evaluated? Without a clear definition and identifiable indicators, it remains unclear what distinguishes the proposed ARCD framework from established pedagogical principles supplemented by digital tools.

 5. What are the qualifications of the AI developer and the two curriculum specialists, and what was their role in validating the framework?

 The Participants section (p. 6) identifies one AI developer-collaborator and two curriculum specialists but provides limited information about their relevant qualifications and professional experience. Could the authors clarify the developer's technical background and whether they developed an AI model, integrated an existing model, or configured an existing application? Similarly, what qualifications and experience did the two curriculum specialists have in ECE curriculum design and evaluation? Were they formally involved in curriculum development, or did they provide general professional opinions? How was the proposed framework evaluated, and what evidence supports its technical and pedagogical validity? The involvement of one developer and two specialists is not inherently insufficient, but their expert opinions should not be treated as independent validation of AI accuracy or curriculum effectiveness. The authors should clearly distinguish expert consultation from systematic validation.

 6. Is the phenomenological design appropriate for the participants' experiences, and how was the integrity of individual experiential accounts preserved?

 The Methods section (p. 6) describes purposive maximum-variation sampling involving six teachers with different levels of professional and AI-integration experience, alongside two curriculum specialists and one AI developer. The authors identify the teachers as the primary phenomenological participants and the other participants as supplementary informants. However, could the authors clarify the shared experiential criterion used to select the six teachers? Did all six have direct experience designing and implementing an AI-responsive curriculum, or did their experiences involve different forms and levels of AI use? How did the researchers ensure that these differences did not compromise the coherence of the phenomenon being investigated?

 The Data Analysis section (pp. 7–8) describes the progression from first order meaning units to second-order themes and third-order composite constructs. However, Table 2 (pp. 8–9) primarily illustrates the aggregation of excerpts into higher-order constructs. Could the authors clarify whether each teacher's account was analysed individually before cross-case synthesis, and how individual experiential trajectories, contradictory accounts, and divergent experiences were preserved? The authors should also explain the methodological rationale for combining an interpretive phenomenological orientation with Giorgi's descriptive phenomenological method. How were the underlying epistemological differences addressed in the analytical process? The use of MAXQDA is not itself a concern; the issue is whether the analytical procedures remain consistent with the stated phenomenological approach.

 7. How does the phenomenological analysis justify the development of the ARCD conceptual framework?

 The title and stated purpose emphasise the development of a conceptual framework, whereas the Methods section (pp. 6–8) describes a phenomenological study of educators' experiences. These approaches are not necessarily incompatible, but the manuscript does not sufficiently explain how the phenomenological findings were transformed into the proposed framework. The authors identify three empirical constructs—Predictive Developmental Tracking, AI-Responsive Curriculum Adaptation, and Deep Learning as Pedagogical Scaffold—and subsequently propose three ARCD principles: Developmental Transparency, Ecological Sensitivity, and Professional Amplification (pp. 9–11). Could the authors clarify the analytical and theoretical steps connecting the three empirical constructs to the three framework principles? Which elements emerged directly from participants' accounts, and which were derived from the literature or the researchers' interpretations?

 Furthermore, the study did not independently validate the AI system or evaluate child-development outcomes. What is the intended epistemic status of the ARCD framework: an interpretive framework grounded in teachers' experiences, a preliminary conceptual model, or a curriculum design model intended for implementation? The authors acknowledge the need for prospective validation (p. 11). How, then, do they justify the framework's claimed contribution and applicability beyond the specific setting studied? The manuscript should clearly distinguish an empirically informed preliminary framework from a validated curriculum model.

Is the work clearly and accurately presented and does it cite the current literature?

No

If applicable, is the statistical analysis and its interpretation appropriate?

Not applicable

Are all the source data underlying the results available to ensure full reproducibility?

Partly

Is the study design appropriate and is the work technically sound?

No

Are the conclusions drawn adequately supported by the results?

Partly

Are sufficient details of methods and analysis provided to allow replication by others?

No

Reviewer Expertise:

Educational Studies, Early Childhood Education, Sexuality Education, Curriculum, Assessment and Evaluation

I confirm that I have read this submission and believe that I have an appropriate level of expertise to state that I do not consider it to be of an acceptable scientific standard, for reasons outlined above.

# Reviewer report: Reviewer response for version 1

**DOI:** 10.5256/f1000research.204905.r508389

**Published:** 2026-08-25

**Authors:** Diah Andika Sari

**Affiliations:** 1 Universitas Muhammadiyah Jakarta, Jakarta, Special Capital Region of Jakarta, Indonesia

**Reviewer Summary & Main Recommendations**

 This manuscript employs a qualitative phenomenological approach and an interpretivist paradigm to explore participants' experiences and perspectives. Overall, the study is conceptually grounded and addresses a relevant topic. However, minor to moderate revisions is recommended before indexing:

**1. Is the work clearly and accurately presented and does it include up-to-date literature?**

**Answer: YES**

 The manuscript is clearly presented and supported by relevant literature. Some important primary and classical references are also used, such as Heidegger (1962) and Bronfenbrenner (1979), which provide a good theoretical foundation. However, most of the other references are from 2018–2022. Since AI in education is developing very rapidly, I suggest adding more recent studies, especially from 2023 onward, related to AI in early childhood education, AI-responsive curriculum, and the impact of AI on young children’s learning and development.

**2. Is the research design appropriate and does the work have a strong technical basis?**

**Answer: YES**

 The qualitative phenomenological approach and interpretivist paradigm are appropriate for the purpose of this study. The approach is suitable for understanding the experiences, perspectives, and meanings of the participants. The use of first-order constructs, second-order constructs, and third-order constructs also provides a useful way to develop a deeper understanding of the phenomenon. The use of purposive sampling and the presentation of the participants’ demographic characteristics are also clearly described. Overall, the research design is appropriate for the objectives of the study.

**3. Are the methods and analysis described in enough detail to allow replication by others?**

**Answer: PARTIALLY**

 The manuscript provides a general explanation of the research approach, participant selection, and data collection. However, the data analysis process could be explained in more detail. In particular, it would be helpful to explain more clearly how the interview data were coded and how the codes were developed into first order, second-order, and third-order constructs. Providing one simple example from the interview data to the coding and final theme would help readers understand the analysis process more clearly.

**4. If relevant, are the statistical analyses and their interpretation appropriate?**

**Answer: NOT APPLICABLE**

 Not applicable, because this study used a qualitative approach and did not use statistical analysis. However, the qualitative analysis could be explained more clearly. For example, the authors could provide a brief example of the coding process or coding framework. If MAXQDA was used, information about the coding structure or audit trail could also be helpful to show how the analysis was conducted.

**5. Is all source data underlying the results available to ensure full reproducibility?**

**Answer: PARTIALLY**

 The manuscript provides several interview excerpts to support the findings. However, the process from the interview data to the codes, themes, and final constructs is not fully shown. Providing a simple coding matrix or codebook would help readers understand how the findings were developed from the original data. It would also be useful to briefly explain how the researchers reflected on their own perspectives during the interpretation process, considering that this is an interpretivist and phenomenological study. Any sharing of raw interview data should also consider participant confidentiality and ethical requirements.

**6. Are the conclusions adequately supported by the results of the research?**

**Answer: YES**

 Yes. The conclusions are generally consistent with the findings presented in the manuscript. The conclusions are also in line with the objectives of the study and do not appear to make claims that go beyond the data.

Is the work clearly and accurately presented and does it cite the current literature?

Yes

If applicable, is the statistical analysis and its interpretation appropriate?

Not applicable

Are all the source data underlying the results available to ensure full reproducibility?

Partly

Is the study design appropriate and is the work technically sound?

Yes

Are the conclusions drawn adequately supported by the results?

Yes

Are sufficient details of methods and analysis provided to allow replication by others?

Partly

Reviewer Expertise:

Early Childhood Education, The impact of gadgets on child development, ECE Management & Curriculum, Play & movement , Dance & Creative Movement

I confirm that I have read this submission and believe that I have an appropriate level of expertise to confirm that it is of an acceptable scientific standard, however I have significant reservations, as outlined above.

# Response

**Published:** 2026-08-27

**Authors:** Herlina Herlina

**Affiliations:** Early Childhood Education Prog, Universitas Negeri Makassar, Makassar, South Sulawesi, Indonesia

We would like to sincerely thank Reviewer 2, Diah Andika Sari, for the careful, constructive, and valuable comments on our manuscript. We greatly appreciate the reviewer’s thoughtful suggestions, which have helped us improve the manuscript’s theoretical foundation, methodological transparency, analytical clarity, and engagement with recent developments in artificial intelligence in early childhood education.

 We have carefully considered all of the reviewer’s comments and have revised the manuscript accordingly. The following point-by-point responses explain how each comment has been addressed and indicate the corresponding changes made in the revised manuscript.

 Once again, we sincerely appreciate the reviewer’s time, expertise, and constructive feedback, which have contributed significantly to strengthening the quality and clarity of our manuscript.

 Comment 1 – Add up-to-date literature

**Reviewer’s Comment:**

 “Since AI in education is developing very rapidly, I suggest adding more recent studies, especially from 2023 onward, related to AI in early childhood education, AI-responsive curriculum, and the impact of AI on young children's learning and development.”

**Response:**

 Thank you very much for this valuable and timely suggestion. We fully agree that the rapidly evolving field of artificial intelligence in education requires the manuscript to engage with recent and relevant evidence, particularly research published from 2023 onward.

 In response to this comment, we have substantially updated the Introduction and Literature Review by incorporating recent empirical studies, systematic reviews, and international policy frameworks addressing artificial intelligence in early childhood education, AI-responsive curriculum development, AI literacy, teacher perspectives, culturally responsive AI, and the potential implications of generative AI for young children's learning and development.

 Specifically, the revised manuscript now incorporates: (1) a 2024 scoping review covering 18 empirical studies of AI in early childhood education across 11 countries; (2) a 2024 study examining kindergarten teachers' perspectives on ChatGPT; (3) a 2024 intervention addressing AI literacy in early childhood education; (4) a 2024 comparative study examining storytelling by parents and ChatGPT with children aged 5–6 years; (5) a 2025 action-research study addressing culturally responsive AI in early childhood teacher education; (6) UNESCO's 2024 AI Competency Framework for Teachers; (7) a 2024 synthesis examining the promises and challenges of AI for human learning; and (8) a 2026 systematic review of generative AI in early childhood education.

 These recent sources have been integrated not merely as additional references but as part of the conceptual synthesis supporting the rationale for an AI-responsive early childhood curriculum. The revised literature review also highlights the continuing limitations of current AI tools in relation to developmental appropriateness, contextual sensitivity, cultural responsiveness, and the need for professional teacher judgment.

**Changes in the Manuscript:**

 The relevant revisions have been incorporated into the following sections:

- Introduction;
- Artificial Intelligence and Deep Learning in Educational Contexts;
- Early Childhood Development and the Limitations of Current AI Tools; and
- Gaps in the Literature and Research Rationale.

The reference list has also been updated to include recent sources, including Chen (2024), Chin et al. (2024), Li and Yu (2025), Miao and Cukurova (2024), Su and Yang (2024a, 2024b), Yan et al. (2024), and Zhang et al. (2026).

 Comment 2 – Appropriateness of the Research Design

**Reviewer’s Comment:**

 The reviewer considered the qualitative phenomenological approach, interpretivist paradigm, purposive sampling, and construct development to be appropriate.

**Response:**

 We sincerely appreciate the reviewer’s positive assessment of the research design. We are pleased that the qualitative phenomenological approach, interpretivist paradigm, purposive sampling strategy, and construct development were considered appropriate for the aims of the study.

 Although no fundamental change to the overall research design was necessary, we have further clarified the methodological roles of the participants and strengthened the description of the analytical process. In particular, the AI tool developer is now explicitly identified as an expert informant who contributed technical and contextual perspectives that supported the interpretation of the educators' accounts.

 The six preschool teachers remain the primary phenomenological participants because their lived experiences constitute the central empirical basis of the study. The two curriculum specialists and one AI tool developer-collaborator provide complementary curricular and technical perspectives that support the interpretation and development of the proposed framework.

**Changes in the Manuscript:**

 The revised manuscript clarifies the participant roles and provides a more explicit explanation of how the technical informant's contribution was distinguished from the primary phenomenological accounts.

 Comment 3 – Insufficient Detail for Replication

**Reviewer’s Comment:**

 “It would be helpful to explain more clearly how the interview data were coded and how the codes were developed into first-order, second-order, and third-order constructs. Providing one simple example from the interview data to the coding and final theme would help readers understand the analysis process more clearly.”

**Response:**

 Thank you for this important methodological suggestion. We agree that greater transparency in the analytical process is essential for enabling readers to understand how the empirical data were transformed into interpretive findings and ultimately into the proposed conceptual constructs.

 Accordingly, we have substantially expanded the Data Analysis section to provide an operational five-step description of the analytical process:

1. familiarization with the data and horizonalization;
2. segmentation of the data into first-order meaning units;
3. clustering of meaning units into second-order interpretive themes;
4. use of imaginative variation to develop third-order composite constructs; and
5. synthesis of textural and structural descriptions.

We have also clarified how interpretations derived from interviews were compared with evidence from observations and reflective documents. In addition, the revised manuscript explains that code definitions, inclusion and exclusion criteria, and analytic decisions were systematically documented throughout the process.

 To provide a concrete illustration, the revised Table 2 traces an interview excerpt from participant P4 concerning the adaptation of an AI-recommended fine-motor activity after the teacher considered a child's family distress. This excerpt was interpreted as the first-order meaning unit **“context overrides or reshapes algorithmic advice,”** which was subsequently developed into the second-order interpretive theme **“contextual interrogation and professional synthesis,”** and finally incorporated into the third-order composite construct **“AI-Responsive Curriculum Adaptation.”**

 This example demonstrates how the analysis moved systematically from participants' lived experiences to interpretive themes and then to the higher-order conceptual framework.

**Changes in the Manuscript:**

 The Data Analysis section has been revised substantially, and Table 2 now presents the complete analytical chain from data excerpt → first-order meaning unit → second-order interpretive theme → third-order composite construct. We also clarified that MAXQDA was used to support data organization and retrieval rather than to automatically generate interpretations or themes.

 Comment 4 – Clarification of MAXQDA Coding Structure and Audit Trail

**Reviewer’s Comment:**

 “If MAXQDA was used, information about the coding structure or audit trail could also be helpful to show how the analysis was conducted.”

**Response:**

 Thank you for highlighting the importance of documenting the coding architecture and audit trail. We agree that providing this information strengthens the methodological transparency and trustworthiness of the study.

 In response, we have clarified that **MAXQDA 2024** was used to organize the source groups, coded segments, code definitions, analytic memos, and cross-source retrieval. The software was used as an organizational and retrieval tool within the research process; it did not independently generate themes or determine the interpretation of the data.

 The revised manuscript also describes the audit trail, which includes dated reflexive memos, successive versions of the codebook, records of code merging and splitting decisions, inclusion and exclusion criteria, links between conceptual constructs and supporting data excerpts, and documentation of member-checking and peer-debriefing decisions.

 These additions make the analytical process more traceable and enable readers to better understand how interpretations were developed and reviewed.

**Changes in the Manuscript:**

 A new paragraph has been added to the Data Analysis section to document the MAXQDA coding architecture and audit-trail procedures. Table 2 also provides a concise, reader-accessible representation of the analytical trace from empirical data to conceptual constructs.

 Comment 5 – Source Data, Codebook, Reflexivity, and Confidentiality

**Reviewer’s Comment:**

 “Providing a simple coding matrix or codebook would help readers understand how the findings were developed from the original data. It would also be useful to briefly explain how the researchers reflected on their own perspectives during the interpretation process. Any sharing of raw interview data should also consider participant confidentiality and ethical requirements.”

**Response:**

 We greatly appreciate this thoughtful recommendation concerning analytical transparency, researcher reflexivity, and ethical data management. We agree that readers should be able to trace the development of the findings while participant confidentiality and ethical obligations are fully maintained.

 In response, we have added **Table 2 as a coding matrix containing eight traceable examples** drawn from interviews, field notes, observations, and a teacher reflective journal. The table demonstrates how selected empirical materials were progressively interpreted through the first-order, second-order, and third-order analytical structure.

 We have also added a new **Researcher Reflexivity and Positionality** subsection. This section explains how the researchers documented their assumptions and perspectives regarding both the potential benefits and risks of AI through reflexive memos. Alternative and disconfirming interpretations were retained during the analytical process rather than being excluded prematurely. In addition, peer debriefing and member checking were used to challenge and refine the research team's interpretations.

 Regarding data sharing, the revised Data Availability statement distinguishes between materials that can be openly shared in de-identified form and confidential raw research records. Because the study involves a single research site and a relatively small participant group, releasing complete interview transcripts or other raw materials could increase the risk of participant re-identification. Therefore, confidential raw materials are not openly released.

 Where appropriate, access to non-public materials may be considered by the corresponding author, provided that such access is consistent with participant consent and the applicable ethics approval.

**Changes in the Manuscript:**

 We have revised Table 2, added the Researcher Reflexivity and Positionality subsection, expanded the Trustworthiness discussion, and revised the Data Availability statement to clarify the conditions governing access to non-public research materials.

 Comment 6 – Conclusions Supported by the Findings

**Reviewer’s Comment:**

 The reviewer considered the conclusions to be consistent with the findings and noted that they do not exceed the data.

**Response:**

 We sincerely thank the reviewer for this positive assessment. We appreciate the recognition that the conclusions are appropriately grounded in the study findings and remain within the evidentiary boundaries of the qualitative data.

 We have retained the principal conclusion of the study while making a minor refinement to the language concerning the novelty of the proposed framework. Specifically, the revised conclusion now characterizes ARCD as an **“empirically grounded, early-childhood-specific framework”** rather than making an absolute claim that it is the first framework of its kind.

 This revision ensures that the contribution of the study is clearly communicated while maintaining an appropriately cautious and evidence-based claim regarding its originality.

**Changes in the Manuscript:**

 The Conclusion section has been revised to moderate the novelty claim while preserving the study's principal contribution.

 We sincerely appreciate Reviewer 2, Diah Andika Sari, for the constructive and insightful comments. The revisions have strengthened the manuscript's methodological transparency, analytical traceability, engagement with current literature, reflexive positioning, and ethical clarity regarding data availability. We believe that these revisions have substantially improved the manuscript while preserving its phenomenological focus and original contribution to the development of an AI-responsive curriculum framework for early childhood education.

 We respectfully submit the revised manuscript for further consideration.

# Reviewer report: Reviewer response for version 1

**DOI:** 10.5256/f1000research.204905.r508395

**Published:** 2026-08-10

**Authors:** Irma Yuliantina

**Affiliations:** 1 Indonesian Coalition for Holistic-Integrative Early Childhood Development, Jakarta, Jakarta, Indonesia; 2 Universitas Panca Sakti Bekasi, Bekasi, West Java, Indonesia

This article explores educators' experiences in designing and implementing an early childhood education curriculum that is responsive to artificial intelligence. Employing a qualitative phenomenological approach, the study develops the AI-Responsive Curriculum Design (ARCD) framework, which encompasses Predictive Developmental Tracking, AI-Responsive Curriculum Adaptation, and Deep Learning as Pedagogical Scaffold. The framework positions AI as a support for teacher decision-making rather than a substitute for educators' professional judgment. However, there is one minor point that needs to be addressed before the article is accepted:Ensure the number of participants is consistent between the abstract and the methods section. The abstract mentions six teachers and two curriculum specialists, totaling eight participants; however, the methods section lists nine participants by including an AI developer. The author needs to clarify this and use the same number of participants consistently. If the AI ​​developer is an additional informant, their status and role should be explicitly stated in the abstract.This discrepancy does not invalidate the research results, but it must be corrected to ensure that the reporting of the methodology is valid and transparent, and does not raise doubts regarding the source of the analyzed data.

Is the work clearly and accurately presented and does it cite the current literature?

Yes

If applicable, is the statistical analysis and its interpretation appropriate?

Yes

Are all the source data underlying the results available to ensure full reproducibility?

Yes

Is the study design appropriate and is the work technically sound?

Yes

Are the conclusions drawn adequately supported by the results?

Yes

Are sufficient details of methods and analysis provided to allow replication by others?

Yes

Reviewer Expertise:

Early Childhood EducationEarly Childhood Education CurriculumArtificial Intelligence in EducationCurriculum Design and DevelopmentAdaptive and Personalized Learning

I confirm that I have read this submission and believe that I have an appropriate level of expertise to confirm that it is of an acceptable scientific standard.

# Response

**Published:** 2026-08-27

**Authors:** Herlina Herlina

**Affiliations:** Early Childhood Education Prog, Universitas Negeri Makassar, Makassar, South Sulawesi, Indonesia

We would like to sincerely thank Reviewer 1, Irma Yuliantina, for the careful, constructive, and valuable evaluation of our manuscript. We greatly appreciate the reviewer’s insightful comments and suggestions, which have helped us improve the clarity, consistency, and methodological transparency of the manuscript.

 We have carefully considered the reviewer’s comments and have revised the manuscript accordingly. Our point-by-point response is provided below.

 Comment 1 – Participant-Count Inconsistency

**Reviewer’s Comment:**

 “Ensure the number of participants is consistent between the abstract and the methods section. The abstract mentions six teachers and two curriculum specialists, totaling eight participants; however, the methods section lists nine participants by including an AI developer. The author needs to clarify this and use the same number of participants consistently.”

**Response:**

 Thank you very much for identifying this important inconsistency. We fully agree that the participant information should be presented consistently and transparently throughout the manuscript.

 In response to the reviewer’s comment, we have carefully revised the manuscript so that the total number of adult participants is consistently reported as **nine**. The participants comprise **six preschool teachers, two curriculum specialists, and one AI tool developer-collaborator**.

 To clarify the different roles of these participants, the six preschool teachers remain the **primary phenomenological participants**, as their lived experiences constitute the central empirical focus of the study. The two curriculum specialists and the AI tool developer-collaborator serve as **expert informants**, contributing complementary curricular and technical perspectives that support the interpretation of the educators’ accounts and the development of the proposed framework.

 We have also explicitly clarified the role of the AI tool developer-collaborator as an additional expert informant who provided technical context for interpreting the teachers’ experiences. This distinction ensures that the inclusion of the technical informant does not obscure the phenomenological focus of the study.

**Changes in the Manuscript:**

 The participant information has been revised consistently across the **Abstract – Method, Methodology – Participants, and Data Collection Procedures** sections. Table 1 has also been checked and remains consistent with **n = 9**.

 The revised wording in the Abstract now states:

 “An interpretive phenomenological study was conducted at TK Harapan Bangsa, Makassar, Indonesia, with nine adult participants: six preschool teachers, two curriculum specialists, and one AI tool developer-collaborator whose expert interview supplied technical context.”

 We believe this revision provides a clearer distinction between the primary phenomenological participants and the additional expert informants while ensuring consistency in participant reporting throughout the manuscript.

 Once again, we sincerely thank Reviewer 1 for identifying this issue and for helping us improve the methodological clarity and consistency of our manuscript.
